# Flux-Enabled Exploration of the Role of Sip1 in Galactose Yeast Metabolism

**DOI:** 10.3389/fbioe.2017.00031

**Published:** 2017-05-24

**Authors:** Christopher M. Shymansky, George Wang, Edward E. K. Baidoo, Jennifer Gin, Amanda Reider Apel, Aindrila Mukhopadhyay, Héctor García Martín, Jay D. Keasling

**Affiliations:** ^1^Biological Systems and Engineering Division, Lawrence Berkeley National Laboratory, Berkeley, CA, USA; ^2^Lawrence Berkeley National Laboratory, Joint BioEnergy Institute, Emeryville, CA, USA; ^3^Department of Chemical and Biomolecular Engineering, University of California Berkeley, Berkeley, CA, USA; ^4^DOE Agile Biofoundry, Emeryville, CA, USA; ^5^BCAM, Basque Center for Applied Mathematics, Mazarredo, Bilbao, Basque Country, Spain; ^6^Department of Bioengineering, University of California Berkeley, Berkeley, CA, USA; ^7^Novo Nordisk Foundation Center for Biosustainability, Technical University of Denmark, Hørsholm, Denmark

**Keywords:** ^13^C metabolic flux analysis, genome-scale models, glucose repression, yeast, metabolomics

## Abstract

^13^C metabolic flux analysis (^13^C MFA) is an important systems biology technique that has been used to investigate microbial metabolism for decades. The heterotrimer Snf1 kinase complex plays a key role in the preference *Saccharomyces cerevisiae* exhibits for glucose over galactose, a phenomenon known as glucose repression or carbon catabolite repression. The *SIP1* gene, encoding a part of this complex, has received little attention, presumably, because its knockout lacks a growth phenotype. We present a fluxomic investigation of the relative effects of the presence of galactose in classically glucose-repressing media and/or knockout of *SIP1* using a multi-scale variant of ^13^C MFA known as 2-Scale ^13^C metabolic flux analysis (2S-^13^C MFA). In this study, all strains have the galactose metabolism deactivated (*gal1*Δ background) so as to be able to separate the metabolic effects purely related to glucose repression from those arising from galactose metabolism. The resulting flux profiles reveal that the presence of galactose in classically glucose-repressing conditions, for a CEN.PK113-7D *gal1*Δ background, results in a substantial decrease in pentose phosphate pathway (PPP) flux and increased flow from cytosolic pyruvate and malate through the mitochondria toward cytosolic branched-chain amino acid biosynthesis. These fluxomic redistributions are accompanied by a higher maximum specific growth rate, both seemingly in violation of glucose repression. Deletion of *SIP1* in the CEN.PK113-7D *gal1*Δ cells grown in mixed glucose/galactose medium results in a further increase. Knockout of this gene in cells grown in glucose-only medium results in no change in growth rate and a corresponding decrease in glucose and ethanol exchange fluxes and flux through pathways involved in aspartate/threonine biosynthesis. Glucose repression appears to be violated at a 1/10 ratio of galactose-to-glucose. Based on the scientific literature, we may have conducted our experiments near a critical sugar ratio that is known to allow galactose to enter the cell. Additionally, we report a number of fluxomic changes associated with these growth rate increases and unexpected flux profile redistributions resulting from deletion of *SIP1* in glucose-only medium.

## 1 Introduction

In the presence of glucose, *Saccharomyces cerevisiae* represses consumption of other carbon sources. This phenomenon, known as glucose repression, involves the repression of genes and pathways involved in respiration (e.g., TCA cycle, etc.), the use of alternative fermentable (e.g., sucrose and galactose) and non-fermentable (e.g., ethanol and acetate) carbon sources, and gluconeogenesis (Zaman et al., 2008; Kayikci, 2015). A better understanding of glucose repression could improve mixed-carbon source fermentation using biomass feedstocks (Apel et al., 2016) and, hence, production of biofuels and other renewable bioproducts (Nielsen et al., 2013).

**Figure 1:**
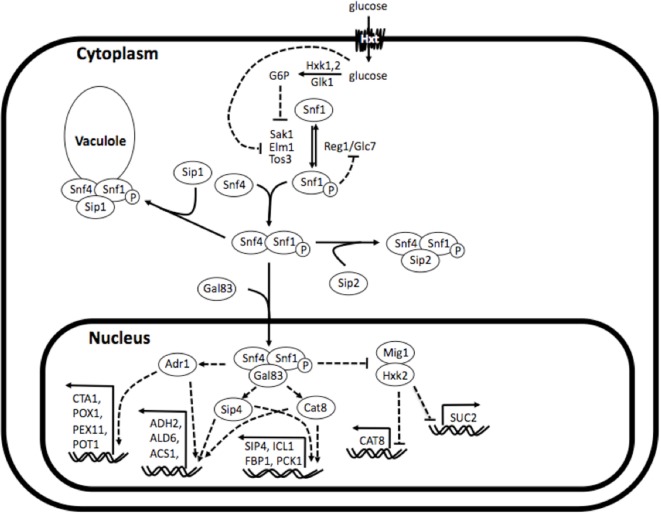
**Simplified depiction of Snf1 kinase complex in and its regulatory interactions**. The Snf1 kinase complex is a heterotrimer consisting of a catalytic *α*-subunit Snf1, regulatory *γ*-subunit Snf4, and one of three *β*-subunits Sip1, Sip2, or Gal83. Under glucose-repressing conditions, these components are found unassembled in the cytosol and, conversely, upon glucose depletion they assemble into all three isoforms of the complex (comprising either Sip1, Sip2, or Gal83). The isoform bound to Gal83 localizes in the nucleus and activates genes responsible for alternate carbon source utilization, the isoform bound to Sip2 remains in the cytosol, and the isoform bound to Sip1 is sequestered in the vacuole. Reconstructed and modified from Zaman et al. (2008).

The Sip1 protein is a component of the Snf1 (sucrose non-fermenting 1) kinase complex, which is central to glucose repression in *S. cerevisiae*. The Snf1 kinase complex is the yeast analog of AMPK (adenosine monophosphate-activated protein kinase), a well studied and highly conserved eukaryotic regulator of cellular uptake of glucose, energy homeostasis, beta-oxidation of fatty acids, etc. (Winder and Hardie, 1999). As depicted in Figure 1, the Snf1 kinase complex is a heterotrimer consisting of a catalytic *α*-subunit Snf1, regulatory *γ*-subunit Snf4, and one of three *β*-subunits Sip1, Sip2, or Gal83. Under glucose-repressing conditions, these components are found unassembled in the cytosol and, conversely, upon glucose depletion they assemble into all three isoforms of the complex (containing either Sip1, Sip2, or Gal83). The isoform bound to Gal83 localizes in the nucleus and activates genes responsible for alternate carbon source utilization, the isoform bound to Sip2 remains in the cytosol, and the isoform bound to Sip1 is sequestered in the vacuole (Zaman et al., 2008).

Little is known about the role of Sip1 under these conditions due to a reported lack of phenotypic difference in growth between wild type and *sip1*Δ mutants (Breslow et al., 2008; Zaman et al., 2008; Zhang et al., 2010).

**Figure 2:**
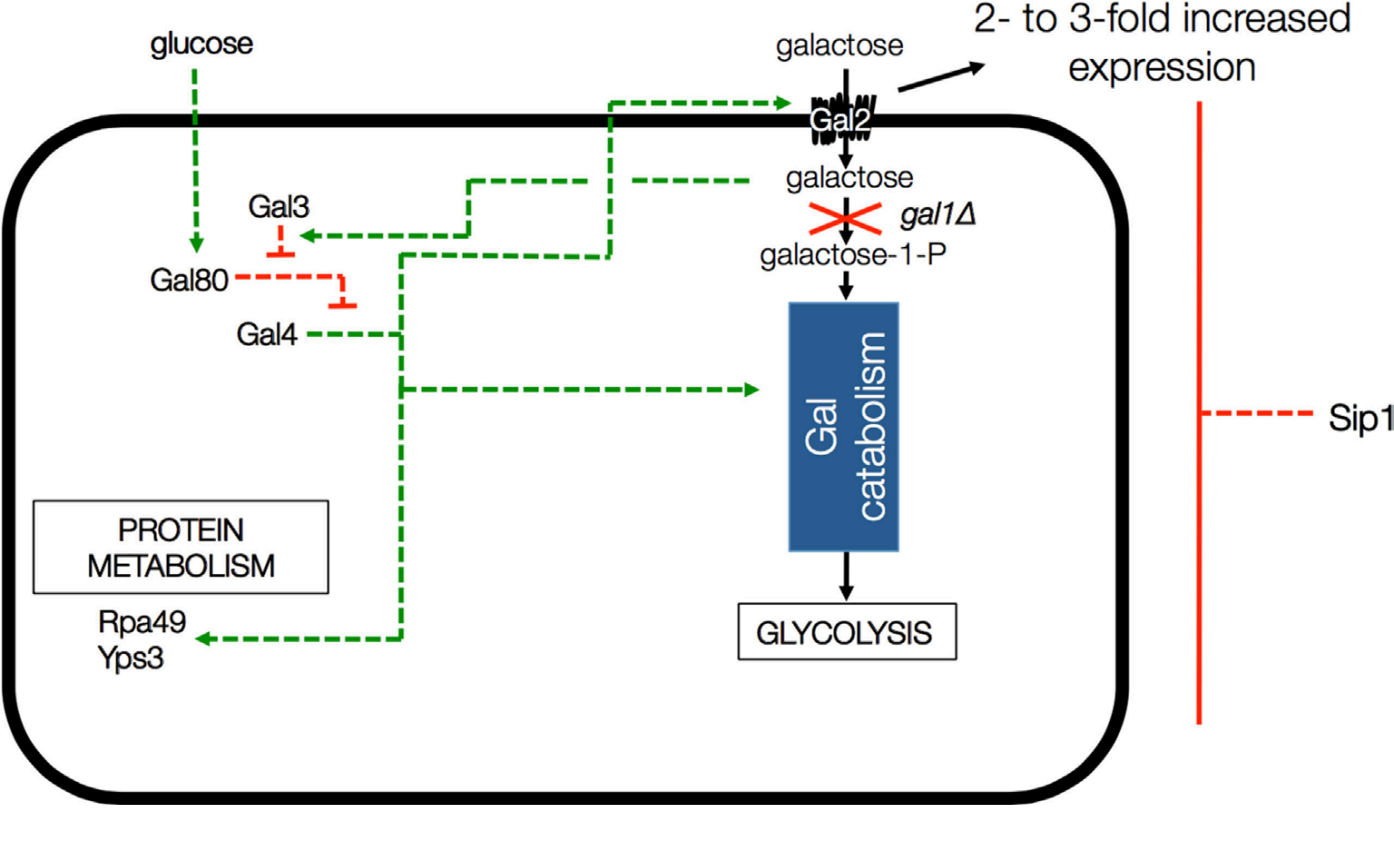
**Depiction of *GAL* gene interactions with Sip1 in a *GAL1* knockout background**. Deletion of *SIP1* is known to increase expression of *GAL2*, the galactose transporter gene, by 2- to 3-fold in glucose-repressing conditions. However, CEN.PK 113-7D is known to be gal2−. The presence of galactose in the cell activates Gal3 which, in turn, represses Gal80. Gal80 represses Gal4 so the full effect is that the deletion of Sip1 derepresses galactose regulation in the cell and, in the end, activates Gal4, a known regulator for many genes, including those involved in alternate carbon source utilization, RNA polymerase III, etc. Green arrows indicate activation and red blunt arrows indicate repression.

One thing that is known about Sip1 is that it is a negative regulator of the galactose utilization system (Mylin et al., 1994), as depicted in Figure 2. Deletion of *SIP1* in yeast is known to increase expression of *GAL2*, the galactose transporter gene, by 2- to 3-fold in glucose-repressing conditions. However CEN.PK113-7D is known to be gal2− (Hansche et al., 1978). The presence of galactose in the cell activates Gal3 which, in turn, represses Gal80. Gal80 represses Gal4 a known regulator for many genes, including those involved in alternate carbon source utilization, RNA polymerase III, etc. (Ideker et al., 2001; Zhang et al., 2010).

In spite of a lack of reported phenotypic difference upon knockout of *SIP1*, in one of our previous studies (Shymansky, 2011), we noticed an increase in specific growth rate upon deletion of this gene in a medium containing both galactose and glucose (though in a different background than reported here: S288c *ura3*Δ *gal1*Δ). We were curious about what shifts in the cell’s metabolic flux profile might be associated with this increase in growth rate. Additionally, we wanted to know how the presence of galactose interacted with this genetic perturbation from a fluxomic perspective.

In this study, we will look into the metabolic effects created by the deletion of *SIP1* by measuring and comparing internal metabolic fluxes, key determinants of microbial physiology. All strains have galactose metabolism deactivated, so as to be able to distinguish glucose repression effects from the impact of galactose metabolism on overall metabolism. Internal metabolic fluxes represent the biomass-normalized activity of metabolic reactions in an organism per hour (Wiechert, 2001; Sauer, 2006). The collection of these metabolic fluxes is known as the fluxome and maps the flow of material through a cell’s metabolism.

Arguably, the two most popular methods of studying flux profiles are Flux Balance Analysis (FBA (Lewis et al., 2012)) and ^13^C metabolic flux analysis (^13^C MFA (Wiechert, 2001; Zamboni, 2011)). FBA uses comprehensive genome-scale metabolic models coupled with experimentally obtained flux bounds and a biological objective (e.g., maximization of growth rate, maximization of ATP production, etc.) to infer flux profiles. ^13^C MFA determines fluxes by combining flux bounds with experimentally measured labeling distributions resulting from ^13^C tracer experiments. Instead of assuming a biological objective, it fits simulated labeling distributions to their measured counterparts. However, it tends to use a less comprehensive metabolic network (García Martín et al., 2015). ^13^C MFA has been used in *S. cerevisiae* to study general batch growth (Maaheimo et al., 2001; Frick and Wittmann, 2005), anaerobic versus aerobic growth (Gombert et al., 2001; Fiaux et al., 2003), varying environmental conditions (Blank and Sauer, 2004), and different gene deletion mutants (Gombert et al., 2001; Blank et al., 2005; Moxley et al., 2009), among others.

A recently published method (García Martín et al., 2015), 2-scale ^13^C MFA (2S-^13^C MFA), combines the strengths of both FBA and ^13^C MFA: comprehensive genome-scale models constrained by ^13^C labeling data without the recourse to a biological objective. This approach models metabolism at two different scales of resolution: the lower scale of resolution constrains fluxes using only stoichiometry for the whole genome-scale model, while a higher resolution scale uses carbon labeling patterns on top of stoichiometry to constrain fluxes for a limited core set of reactions anticipated to carry most of the flux. A critical assumption is that most core reaction’s metabolites are not heavily affected by peripheral metabolism, an assumption that is routinely used in ^13^C MFA and can describe experimental data satisfactorily (Antoniewicz et al., 2007; Schaub et al., 2008; Moxley et al., 2009). This assumption is tested through a External Labeling Variability Analysis (ELVA), and the core set of reactions can be changed as needed to guarantee self-consistency. The results for the core reactions for 2S-^13^C MFA are equivalent to those for ^13^C MFA, but 2S-^13^C MFA extrapolates the constraints induced by the ^13^C labeling data to a genome-scale model. The advantage of 2S-^13^C MFA versus using full genome-scale carbon labeling tracking is that it is a general approach that can be used even if carbon transitions are not available for the full genome-scale model [as is the case for *S. cerevisiae* (Gopalakrishnan and Maranas, 2015)]. Furthermore, it can easily leverage information from previous ^13^C MFA studies (Ghosh et al., 2016).

In this study, we performed an exploratory analysis, via 2S-^13^C MFA, of a set of GAL1^−^ strains with (base strain) and without (*sip1*Δ knockout) an intact *SIP1* gene, similar to those from our previous work (Shymansky, 2011), but in the more industrially relevant CEN.PK113-7D background. We characterized growth and flux profiles for both strains in both glucose-only and mixed glucose/galactose medium and used the detailed information provided by flux profiles to gain insight into the ensuing metabolic changes. The point of this study was to investigate in detail the surprising effect of a change in growth when adding galactose during glucose repression conditions, when galactose should have been ignored by the cell. We use flux analysis because fluxes describe how mass and energy are distributed in cell metabolism and growth rate changes are modeled in genome-scale models as changes in flux for biomass reactions (i.e., reactions that codify all metabolites needed for creating a new cell). 2S-^13^C MFA is unique because it measures fluxes for genome-scale models in an accurate and comprehensive manner, being able to map all reactions encoded in the genome. In this way, we can study in detail how metabolism has been affected by a perturbation that should not have affected it.

## 2 Materials and Methods

### 2.1 Media and Culturing Conditions

Media used in this study, along with their component concentrations, are listed in Table S1 in Supplementary Material. For both genetic manipulations and growth and tracer experiments, all strains were grown in non-baffled shake flasks at 30°C at 200 rpm in either minimal glucose medium (Min), minimal glucose medium with galactose (Min + Gal), YPD, or Sc-Ura. All strains were stored in 20% glycerol stocks at −80°C. Labeled media used 80% 1-^13^C glucose and 20% U-^13^C glucose at the same total concentration of 2% glucose. Exponential-phase cells were obtained by streaking from −80°C glycerol stocks on YPD plates, incubating 5-mL YPD cultures overnight, inoculating into 40 mL of unlabeled media of the final desired composition, and grown until exponential phase (usually 0.6–0.9 OD_600_).

### 2.2 Strain Construction

Prototrophic base (base) and mutant (sip1Δ) *S. cerevisiae* strains were constructed in a haploid CEN.PK113-7D (*MATa URA3 HIS3, LEU2 TRP1 MAL2-8c SUC2*) (Entian and Kötter, 2007) background containing a *URA3* knockout. All strains used in this study are listed in Table 1 with their strain designations, parent strain, genotype descriptions, and Inventory of Composable Elements (ICE) reference numbers (Ham et al., 2012) (https://public-registry.jbei.org). All knockouts were constructed via a near-markerless loxP/Cre recombinase strategy (Güldener et al., 1996) and PCR verified. Briefly, each knockout cassette was amplified from a *loxP-kanMX-loxP* plasmid, pUG6 (Güldener et al., 1996), using the primers listed in Table 2, transformed into yeast using a heat shock method (Agatep et al., 1998), selected on YPD + G418 (geneticin) plates, and PCR verified using primers listed in Table 3. In order to loop out the *kanMX* marker, a Cre recombinase plasmid was transformed in the resulting *kanMX* cassette integrants and plated on selective medium. The selective plate varied depending on the knockout. For knockout of *SIP1*, the Cre recombinase promoter was Gal1p [pSH47 (Güldener et al., 1996)] and selection occurred on pSH47 plates. A different plasmid was necessary for knockout of *GAL1*, since the strain could not grow on galactose. We opted for expression of the Cre recombinase under a constitutive *TEF1* promoter. This new plasmid, pCMS1, was constructed via yeast cloning using *Sac*I and *Xba*I digested pSH47, to excise Gal1p, and Tef1p amplified with regions homologous to the cut ends and subsequent selection on Sc-Ura plates. All loop-outs were PCR verified using the same verification primers in Table 3 and pCMS1 was sequence verified. Cre recombinase plasmids were cured by streaking on YPD plates, growing overnight in liquid YPD medium, streaking to single colonies on YPD plates, simultaneously streaking on YPD and Sc-Ura plates, and glycerol storing YPD plate colonies whose corresponding Sc-Ura colonies did not grow. Prototrophic final base and mutant strains were completed via transformation of a *URA3* plasmid, pRS416 (Sikorski and Hieter, 1989).

**Table 1:** **List of strains, their parents, and genotypes**.

Strain name	Parent strain	Description	ICE part ID
CPU	CEN.PK113-7D	CEN.PK113-7D *ura3*Δ	JBx_026749
CMSY3	CPU	CPU *SIP1::loxP-kanMX-loxP*	JBx_026263
CMSY4	CMSY3	CPU *sip1*Δ	JBx_026264
CMSY5	CPU	CPU *GAL1::loxP-kanMX-loxP*	JBx_026208
CMSY7	CMSY5	CPU *gal1*Δ	JBx_026210
CMSY6	CMSY4	CPU *sip1*Δ *GAL1::loxP-kanMX-loxP*	JBx_026209
CMSY8	CMSY6	CPU *sip1*Δ *gal1*Δ	JBx_026211
base	CMSY7	CPU *gal1*Δ [pRS416]	JBx_026749
sip1Δ	CMSY8	CPU *sip1*Δ *gal1*Δ [pRS416]	JBx_026750

*Details are available in the public instance of the JBEI public registry (Ham et al., 2012) (https://public-registry.jbei.org)*.

**Table 2:** **Deleted genes and corresponding templates and forward/reverse primers used to construct knockout cassettes**.

Knocked out gene	Template	F-primer	R-primer
*GAL1*	pUG6	AAAAATTGTTAATATACCTCTAACGTCAAGGAGAAAAAagctgaagcttcgtacgc	GTAGAAAAAAATGAGAAGTTGTTCTGAACAAAGTAAAAAAAAGAAGTATACcataggccactagtggatctg
*SIP1*	pUG6	CTGACATCTTGGAAAGTTGAACTGTCATATTATATAGTTGTTGCAGCCGCCagctgaagcttcgtacgc	AGAAAAAAATTGAATTAATAGAGTTCGTGAGAATCATTGCGAATTGAGAaggccactagtggatctg

*Uppercase indicates homologous flanking regions and lowercase designates regions binding to pUG6 plasmid*.

**Table 3:** **Primers used to PCR verify specific gene deletions**.

Knocked out gene	F-primer	R-primer
*GAL1*	TTATTTCTGGGGTAAT	TCCCTGTGTTTCAA
	TAATCAGCGAAG	AGTTTGTGG
*SIP1*	GCACTTCTTTTTTTGC	CGTTCTAGGAGCCA
	GTGTGG	TAGGAATC

### 2.3 Growth Characterization and Tracer Experiments

Cell and extracellular metabolite concentrations were monitored during exponential phase in strain characterization batch experiments. These data were necessary to calculate extracellular fluxes and specific growth rates used to mathematically constrain flux profile inference. Exponentially growing cells, obtained as described in the Media and culturing conditions section, were used to inoculate, in quadruplicate, the final 40-mL shake flask cultures to achieve exponential growth the following morning. Optical density was monitored at 600 nm via UV–VIS, and 200 µL samples were spin-filtered and kept at −20°C for subsequent HPLC analysis.

Exponentially growing cells (obtained as described above) were used to inoculate 40 mL labeled shake flask cultures in quadruplicate and monitored via UV–VIS. To prevent changes in intracellular metabolite labeling patterns, 1 mL mid-log (~0.75 OD_600_) samples were taken, spun down (1 min, max speed, 4°C), immediately quenched with 300 µL ice-cold methanol, and kept at −80°C.

### 2.4 Labeled Biomass Sample Processing

Labeling distributions were obtained from processed labeled biomass samples for intracellular 3-phospho-d-glycerate (3 pg), alanine (Ala), arginine (Arg), asparagine (Asp), glutamine (Gln), glutamate (Glu), isoleucine (Ile), leucine (Leu), lysine (Lys), phenylalanine (Phe), threonine (Thr), tyrosine (Tyr), valine (Val), citrate (cit_m), fructose 1,6-bisphosphate (fdp), and succinate (succ_m). Succinate and citrate were assumed to be mitochondrial, while the rest are assumed to be cytosolic as has been done in previous studies (Moxley et al., 2009). The closeness of fit of these data with corresponding simulated values provided a measure of the quality of inferred flux distributions. Labeled biomass samples were mixed with 300 µL ice-cold chloroform and 150 µL ice-cold water, spun down, bead-beated with 500 µL acid-washed beads (10 times, 10 s, 1 min on ice between sonication bursts) in 1.7 mL screw cap tubes, the bottom of the tube was punctured with a needle, and the beads were separated from the solution by spinning (1 min, 1,000 *g*, 4°C) into a 2-mL collection tube. The aqueous layer was filtered (3 k MW cut-off (Amicon), 1.5 h, 13,000 *g*, 4°C), mixed with 1 mL ice-cold H_2_O, and snap frozen in liquid nitrogen. Three holes were punched in the tube cap, and the samples were lyophilized for 24 h. Lyophilized samples were resuspended in 40 µL 50/50 MeOH/H_2_O, and stored at −80°C. Samples were analyzed to obtain intracellular amino acid and non-amino acid labeling data via LC-MS as previously described (Bokinsky et al., 2013; Weaver et al., 2015).

### 2.5 Extracellular Concentration Determination

Extracellular concentrations for glucose, galactose, ethanol, glycerol, succinate, lactate, acetate, and formate were measured via HPLC. These concentrations, along with corresponding culture specific growth rates, were necessary to calculate extracellular fluxes. The 4 mM H_2_SO_4_ eluent flowed through a 1200 Series HPLC (Agilent Technologies, CA) outfitted with UV and refraction index detectors and an Organic Acid Analysis Column (Aminex HPX-87H Ion Exclusion Column, 300 mm 7.8 mm, 50°C, Cat# 125-0140 Bio-Rad, CA, USA) at a rate of 0.6 mL/min. Standards were used to identify metabolite retention times and sample concentrations.

### 2.6 Extracellular Flux and Intracellular Labeling Input Calculations

Extracellular fluxes and specific growth rates were derived from extracellular concentration and optical density time curves. Their means and standard deviations were used to constrain exchange fluxes for consumed and excreted metabolites and biomass fluxes during flux profile inference. Flask-specific maximum specific growth rates were determined from the slope of lnOD versus time data in manually determined linear ranges. The same time points were used with corresponding concentration data to calculate extracellular fluxes. The extracellular flux of metabolite *p, ν_p_*, is give by equation (1). *M_p_* is the corresponding molecular weight of metabolite *p, α* is the conversion factor between OD_600_ and cell mass concentration in grams of dry cell weight per liter (gDcW/L), and $d\bar{C}p/dOD$ is the slope of the concentration of metabolite *p* versus OD_600_. The value of *α* was taken to be 0.7742 based on multiple in-house experiments (data not shown). The average plus and minus the corresponding standard deviation was used to constrain all extracellular fluxes and specific growth rates.

(1) $$\nu_{p}=1,000\;\frac{\mu }{M_{p}\alpha }\;\frac{d\bar{C}p}{dOD},$$

Units are in mmol/gDcW/hr (hence the 1,000 factor).

### 2.7 Flux Profile Inference *via* 2S-^13^C MFA

Flux profiles were inferred from growth and tracer experiment data using 2-scale-^13^C metabolic flux analysis (2S-^13^C MFA) (García Martín et al., 2015). These fluxes, along with specific growth rates and extracellular fluxes were used to characterize the relative effects of the presence of galactose and/or knockout of *SIP1*. 2S-^13^C MFA was chosen over ^13^C MFA for its ability to describe metabolism more comprehensively through genome-scale models, iMM904 (Mo et al., 2009) in this case. The means and standard deviations for strain/condition-specific extracellular fluxes, intracellular metabolite LC-MS fractional labeling distributions, specific growth rates, and feed glucose labeling were used as inputs for the code included as Supplementary Material. The carbon transitions differed for each strain/condition pair as demanded by the ELVA requirements [see Figure 4 and supp. Fig 22 in García Martín et al. (2015)]. Starting from a base core reaction network, carbon transition information was added to reactions (i.e., the reaction was added to the core network) with the largest flux from non-core metabolism to core metabolism. This was performed iteratively until computational errors in the ELVA plot were minimal. This test guaranteed that labeling from outside the core model [e.g., labeling from *CO*_2_ and formate (Gopalakrishnan and Maranas, 2015)] had a minimal impact in the measured labeling patterns. A final ^13^C Flux Variability Analysis (^13^C FVA) was used to find the maximum and minimum values of each flux compatibles with the experimental data [see (García Martín et al., 2015) for more details]. Our code uses the CONOPT Solver in a GAMS framework to perform the ^13^C MFA step using 30 initial flux starting points.

Confidence intervals and goodness-of-fit were calculated as in García Martín et al. (2015). Briefly, the usual ^13^C MFA goodness-of-fit estimates based on the chi square distribution, such as those proposed by Antoniewicz et al. (2006), are not applicable to 2S-^13^C MFA using genome-scale models [see page 24 in García Martín et al. (2015)]. This problem was surmounted by incorporating the goodness-of-fit considerations in the confidence intervals: good fits produce narrow flux confidence intervals (good flux resolution) and bad fits produce large confidence intervals (bad flux resolution). These confidence intervals are calculated by finding the maximum and minimum values of each flux compatible with experimental error [page 26 in García Martín et al. (2015)]. The experimental error for each *m* in the Mass Distribution Vector (MDV) was the maximum of the instrument error and the difference of the best fit computational labeling with the experimental labeling [equation (23) in García Martín et al. (2015)]. Hence a bad fit provides a large experimental error and begets large flux confidence intervals and less flux resolution, and a good fit provides narrower confidence intervals and better flux resolution.

## 3 Results

### 3.1 Growth Rates

**Figure 3:**
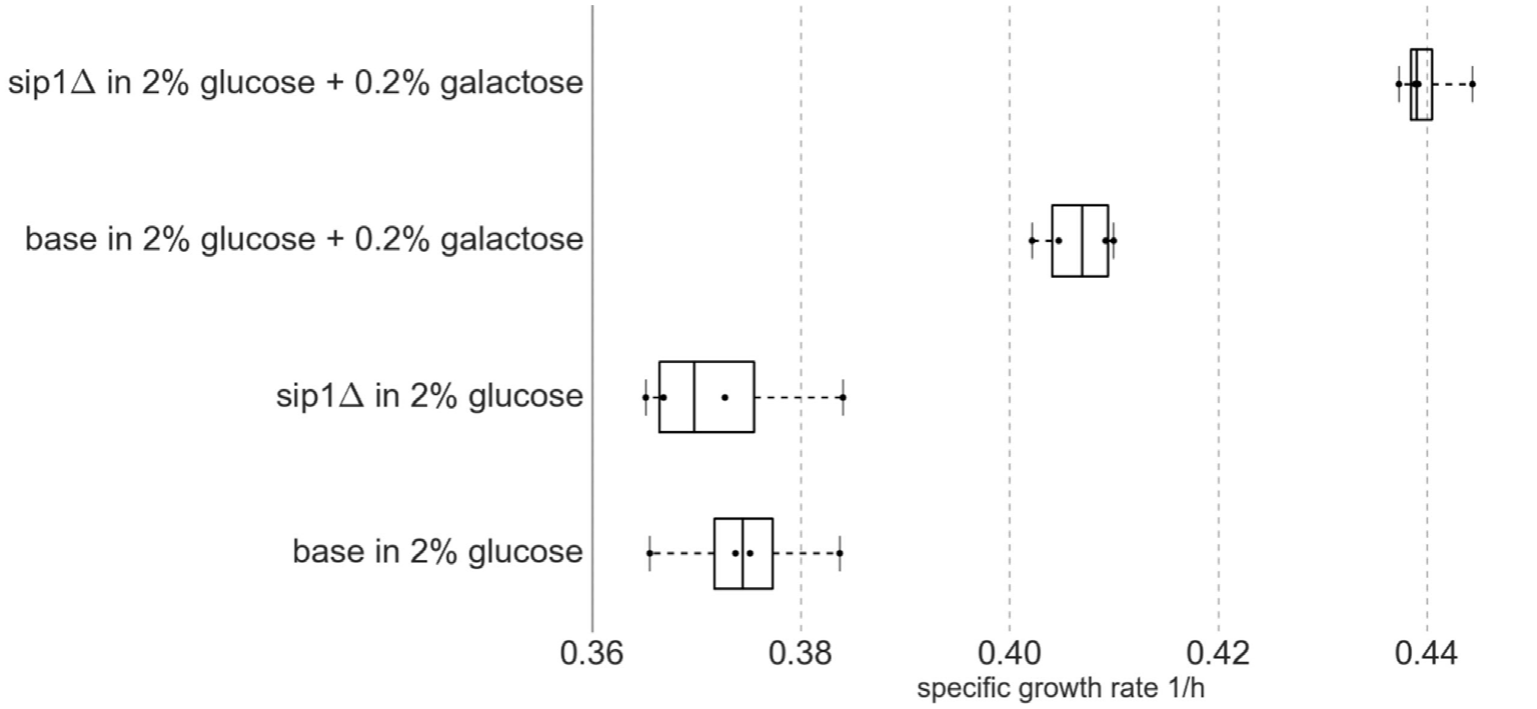
**Box-and-whisker plot of maximum specific growth rates for four biological replicates (n = 4) per strain/condition pair**. Middle box line represents median and edges represent first and third quartiles. Whiskers represent range of data. All data points are displayed. Knockout of *SIP1* had no effect on growth in glucose-repressing minimal medium. Surprisingly, under glucose-repressing conditions, the presence of galactose in the medium had an effect on growth rate, one that was intensified by the *SIP1* knockout.

The presence of galactose in the medium increased the maximum specific growth rate for both base and *sip1*Δ mutant strains. Additionally, the deletion of *SIP1* only resulted in a growth rate increase when galactose was present in the medium. Average maximum specific growth rates for the four strain/condition combinations are presented in Table 4 and a corresponding box-and-whisker plot is presented in Figure 3. An ~8.5% increase was observed when the base strain was grown in medium with supplemented galactose instead of glucose-only medium (base in 2% glucose versus base in 2% glucose + 0.2% galactose). Similarly, the *sip1*Δ mutant grew ~18% faster in galactose-supplemented medium relative to that without (sip1Δ in 2% glucose versus sip1Δ in 2% glucose + 0.2% galactose). The *sip1*Δ mutant grew ~8% faster than the base strain in mixed glucose/galactose medium (base in 2% glucose + 0.2% galactose versus sip1Δ 2% glucose + 0.2% galactose). Consistent with the literature (Breslow et al., 2008; Zhang et al., 2010), no change in maximum specific growth rate was observed between the base and *sip1*Δ mutant strains in glucose-only medium (base in 2% glucose versus sip1Δ in 2% glucose).

**Table 4:** **Extracellular fluxes, *ν*_metabolite_, or maximum specific growth rate, *μ*, means plus or minus the standard deviation for all strain/condition pairs**.

*ν*_metabolite_ or *μ*	U	S	UG	SG
*μ*	0.375 ± 0.007	0.372 ± 0.009	0.407 ± 0.004	0.440 ± 0.003
Glucose	65.71 ± 14.50	36.60 ± 3.47	32.33 ± 4.72	64.06 ± 10.26
Acetate	0.73 ± 0.08	0.62 ± 0.11	1.12 ± 0.09	1.71 ± 0.22
Ethanol	20.62 ± 1.84	5.87 ± 3.52	15.96 ± 2.30	20.56 ± 2.84
Formate	−0.016 ± 0.03	n.d.	n.d.	n.d.
Glycerol	1.49 ± 0.10	1.30 ± 0.21	1.71 ± 0.32	3.00 ± 0.61
Succinate	n.d.	n.d.	n.d.	n.d.
Lactate	n.d.	n.d.	n.d.	n.d.

*Fluxes are in mmol/gDcW/h and growth rates are in 1/h. Strain/condition pair designations U, S, UG, and SG refer to base in 2% glucose, sip1Δ in 2% glucose, base in 2% glucose + 0.2% galactose, and sip1Δ in 2% glucose + 0.2% galactose, respectively*.

### 3.2 Extracellular Fluxes

Neither the presence of galactose nor knockout of *SIP1* resulted in any clear pattern in the extracellular fluxes. The means and standard deviations for all monitored extracellular metabolites for all strain/condition pairs can be found in Table 4. The extracellular flux input ranges, as the mean plus or minus one standard deviation, for strain condition pairs base in 2% glucose, sip1Δ in 2% glucose, base in 2% glucose + 0.2% galactose, and sip1Δ in 2% glucose + 0.2% galactose are presented in Table 4. The *sip1*Δ mutant consumed glucose and excreted ethanol at ~44% and ~72% lower rates, respectively, than the base strain in glucose-only medium (base in 2% glucose versus sip1Δ in 2% glucose). The *sip1*Δ mutant in glucose/galactose medium relative to the parental strain (base in 2% glucose + 0.2% galactose vs sip1Δ in 2% glucose + 0.2% galactose) exhibited a ~98% increase in absolute glucose flux, a ~70% increase in absolute ethanol flux, and a ~54% increase in acetate. Addition of 0.2% galactose to the medium of the base strain (base in 2% glucose vs base in 2% glucose + 0.2% galactose) resulted in a decrease in absolute glucose flux of ~51% and an increase of ~55% for acetate flux. Addition of galactose to the medium of the mutant strain (sip1Δ in 2% glucose vs sip1Δ in 2% glucose + 0.2% galactose) resulted in a ~75% increase in absolute glucose flux, a ~250% increase in ethanol flux, a ~130% increase in glycerol flux, and a ~175% increase in acetate flux. Lactate, formate, and succinate were not detected in any strain/condition pair, hence their extracellular fluxes were considered zero. All other absolute fluxes were the same for all strain/condition pairs within error. The presence of galactose in the medium of the *sip1*Δ mutant appeared to restore the ethanol flux to its value before the gene knockout (sip1Δ in 2% glucose versus sip1Δ in 2% glucose + 0.2% galactose), within error.

In spite of this lack of clear patterns, we will see below that the addition of ^13^C labeling information in the context of the genome-scale model results in noticeable patterns for the intracellular fluxes.

### 3.3 Fits and ELVA Plots

**Figure 4:**
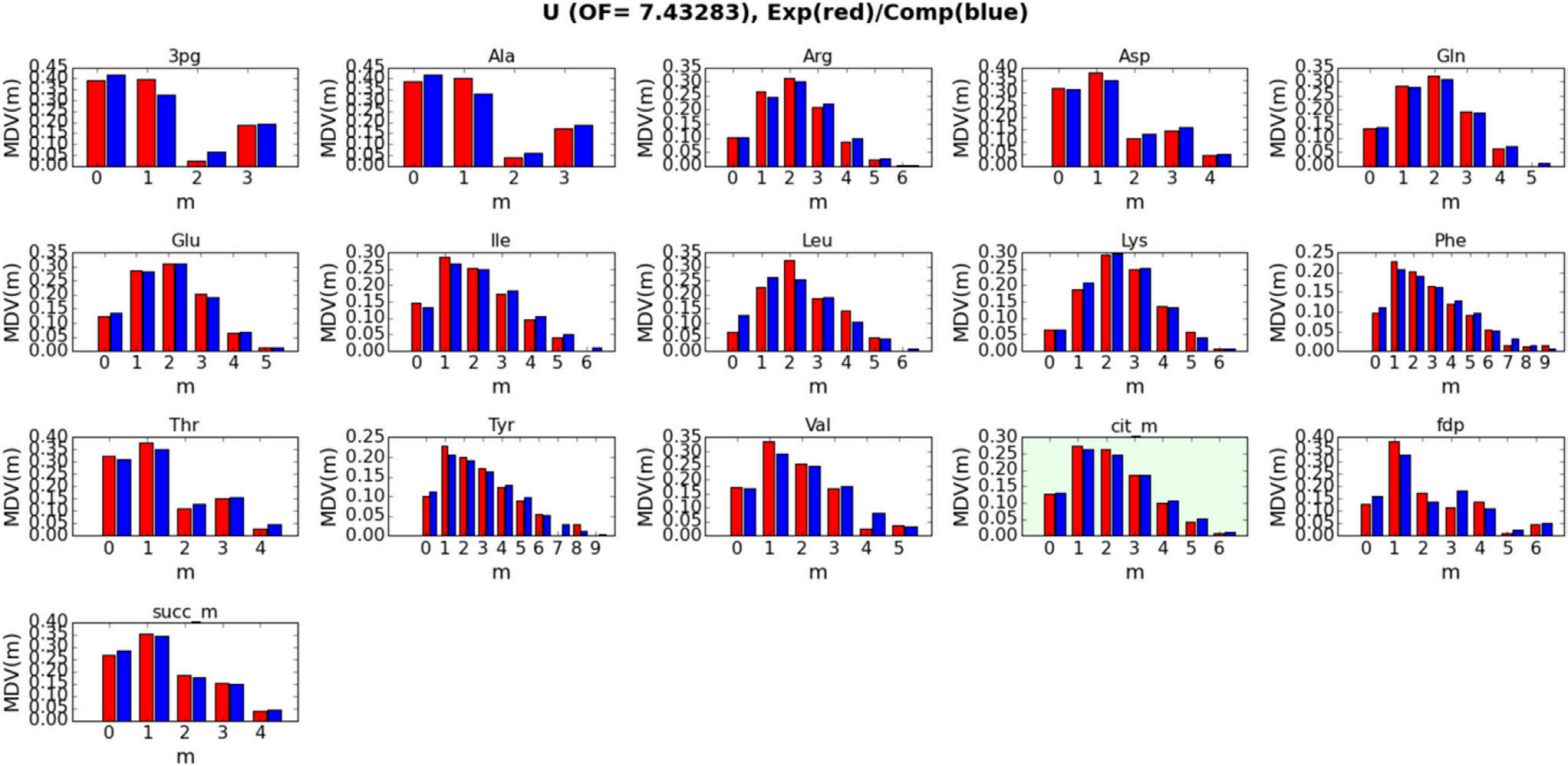
**Detailed fits between simulated (blue bars) and measured (red bars) intracellular metabolite labeling distributions for base in 2% glucose (U) (other strain/conditions can be found in Supplementary Material)**. The correspondence between simulated and measured labeling distributions validates the model. The green box corresponds to a metabolite that was not included in calculating the fluxes (i.e., the fit): its computational labeling distribution values were derived from the fluxes obtained from all the other metabolites, further validating the model. Confidence intervals and goodness-of-fit considerations are addressed in Materials and Methods (section 2.7).

Detailed fits between simulated and measured LC-MS data for metabolites 3-phospho-d-glycerate (3 pg), alanine (Ala), arginine (Arg), asparagine (Asp), glutamine (Gln), glutamate (Glu), isoleucine (Ile), leucine (Leu), lysine (Lys), phenylalanine (Phe), threonine (Thr), tyrosine (Tyr), valine (Val), citrate (cit_m), fructose 1,6-bisphosphate (fdp), and succinate (succ_m) are displayed in Figure 4 for strain/condition pair base + 2% glucose and in Figures S1–S3 in Supplementary Material for sip1Δ in 2% glucose, base in 2% glucose + 0.2% galactose, and sip1Δ in 2% glucose + 0.2% galactose, respectively. We decided to exclude the labeling data for citrate from the fitting to test how well the fluxes fit by the other metabolites could predict its labeling. Predicted citrate labeling closely matches that measured. The ELVA plots (García Martín et al., 2015), used to confirm that reactions external to the core set do not significantly contribute to the core labeling, for all strain/condition pairs are presented in Figure S4 in Supplementary Material. Strain/condition pair sip1Δ in 2% glucose + 0.2% galactose exhibited more variability in both its measured and simulated data errors. Also, sip1Δ in 2% glucose + 0.2% galactose had a somewhat worse fit. The whole flux profiles corresponding to these strain/condition pair ELVA plots are displayed in Figures S5–S8 in Supplementary Material for base in 2% glucose, sip1Δ in 2% glucose, base in 2% glucose + 0.2% galactose, and sip1Δ in 2% glucose + 0.2% galactose, respectively. All values are normalized to the absolute glucose uptake rate. As indicated in the legend in the lower-right of the figure, differently colored small arrows indicate the use of particular cofactors. Cofactors displayed are NADPH, NADH, ATP, GLN-L, AKG-L, NADP, NAD, ADP, GLU-L, ACCOA (acetyl-CoA), FOR (formate), CO_2_, AMP, and CoASH. Arrows pointing toward the main black reaction arrow indicate the cofactor is a reactant and vice versa.

### 3.4 Pentose Phosphate Pathway Activity

**Figure 5:**
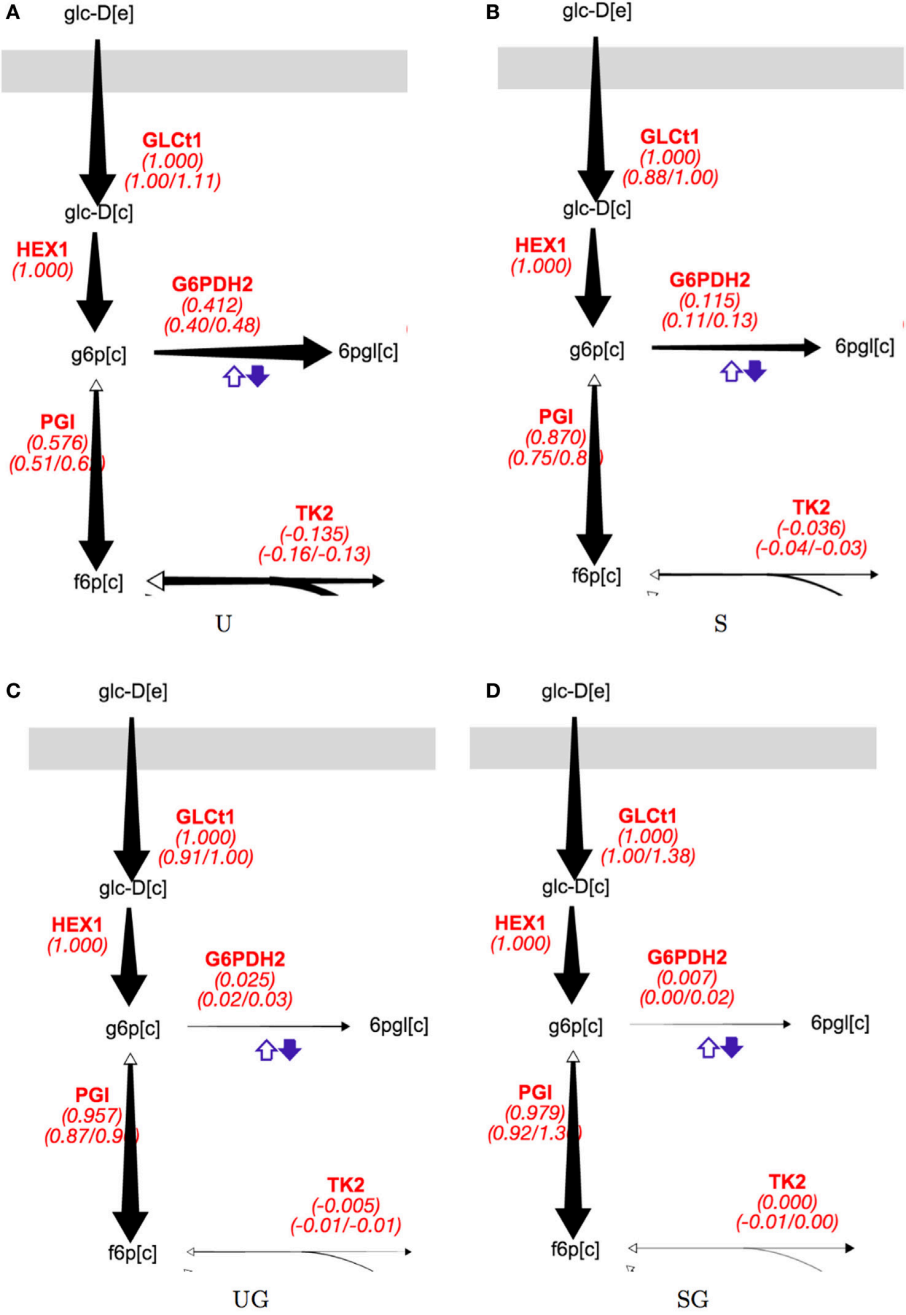
**Split of flux between glycolysis and the PPP for all strain/condition pairs**. Strain/condition pair designations U, S, UG, and SG refer to base in 2% glucose (**A**), sip1Δ in 2% glucose (**B**), base in 2% glucose + 0.2% galactose (**C**), and sip1Δ in 2% glucose + 0.2% galactose (**D**), respectively. Flux values and their absolute ranges obtained from the ELVA are presented in Table 5. PPP flux decreases markedly for both the base and *sip1*Δ mutant when in the presence of galactose (U vs UG and S vs SG) in spite of the glucose-repressing conditions. The size of the arrow corresponds to the reaction flux. Names in red are reaction names used according to the BIGG data base (King et al., 2015). The middle value for each red reaction label is the flux value corresponding to best fit to measured data; the left and right values below are the minimum and maximum values of the flux compatible with the labeling data (from the ^13^C FVA). For a description of colorful cofactor arrows, see the Fits and ELVA plots subsection in the Results section. Flux maps for the other reactions can be found in the Supplementary Material.

The presence of galactose appeared to greatly reduce pentose phosphate pathway (PPP) activity. The split between glycolysis and the PPP for strain/condition pairs base in 2% glucose, sip1Δ in 2% glucose, base in 2% glucose + 0.2% galactose, and base in 2% glucose + 0.2% galactose are displayed in Figure 5 and individual flux values and their absolute ranges from the ELVA are presented in Table 5. When switching from Min to Min + Gal the base strain’s PPP activity reduced by about 94% (base in 2% glucose versus base in 2% glucose + 0.2% galactose). Analogously, the PPP flux for the *SIP1* null mutant decreased a similar ~93% when galactose was present in the medium (sip1Δ in 2% glucose versus sip1Δ in 2% glucose + 0.2% galactose).

**Table 5:** **Flux values corresponding to the split between glycolysis and the PPP, visualized in Figure 5, and that between cytosolic aspartate and malate synthesis, visualized in Figure 6**.

	Glycolysis/PPP split			Aspartate/malate split		
Strain/condition	HEX1	G6PDH2	PGI	PC	ASPTA	MDH
U	1.00	0.41 (0.40/0.48)	0.58 (0.51/0.60)	0.43 (0.38/0.51)	−0.42 (−0.48/−0.37)	0.00 (−0.01/0.01)
S	1.00	0.12 (0.11/0.13)	0.87 (0.75/0.9)	0.28 (0.27/0.35)	−0.25 (−0.32/−0.24)	0.00 (−0.05/0.02)
UG	1.00	0.03 (0.02/0.03)	0.96 (0.87/1.0)	0.47 (0.39/0.47)	−0.09 (−0.11/−0.09)	−0.36 (−0.46/−0.27)
SG	1.00	0.01 (0.00/0.02)	0.98 (0.92/1.3)	0.37 (0.02/0.60)	−0.08 (−0.18/−0.01)	−0.28 (−0.52/0.02)

*Strain/condition pair designations U, S, UG, and SG refer to base in 2% glucose, sip1Δ in 2% glucose, base in 2% glucose + 0.2% galactose, and sip1Δ in 2% glucose + 0.2% galactose, respectively. HEX1, G6PDH2, and PGI refer to the hexokinase, glucose 6-phosphate dehydrogenase, and glucose-6-phosphate isomerase reactions, respectively. PC, ASPTA, and MDH refer to the pyruvate carboxylase, aspartate transaminase, and malate dehydrogenase reactions, respectively. Flux values and their minimum and maximum values obtained from the ELVA are provided. All fluxes are unitless and normalized to the glucose consumption rate*.

### 3.5 Inactive Glyoxylate and TCA Cycles

Both the TCA cycle and the glyoxylate shunt, as expected from glucose repression, appeared to be almost completely repressed across all strain/condition pairs (~1%). This is mostly consistent with the ^13^C MFA literature, which indicates a small amount of activity (usually about 1–2% of total glucose consumption flux) in glucose-repressing conditions for CEN.PK113-7D (Gombert et al., 2001; Maaheimo et al., 2001; Blank and Sauer, 2004; Blank et al., 2005). Fluxes surrounding mitochondrial import and export for strain/condition pairs base in 2% glucose, sip1Δ in 2% glucose, base in 2% glucose + 0.2% galactose, and sip1Δ in 2% glucose + 0.2% galactose are displayed in Figures S9–S12 in Supplementary Material, respectively.

### 3.6 Mitochondrial Import/Export and Branched-Chain Amino Acid Generation

The presence of galactose in the medium for either the base or mutant strains, both with galactose metabolism deactivated, appears to greatly increase mitochondrial activity (Figures S9–S12 in Supplementary Material). Neither strain exhibits mitochondrial import of malate or pyruvate in glucose-repressing conditions. Addition of 0.2% galactose to the medium of both strains (base in 2% glucose vs base in 2% glucose + 0.2% galactose and sip1Δ in 2% glucose vs sip1Δ in 2% glucose + 0.2% galactose) resulted in a dramatic import of malate. This malate is fed through the NADP-dependent malic enzyme to generate mitochondrial pyruvate. Similarly, pyruvate import is activated. Finally, this pyruvate generation flux is directed toward branched-chain amino acids, particularly valine. It should be noted, however, that our ability to compare mitochondrial fluxes of the mutant strain in mixed-carbon medium (sip1Δ in 2% glucose + 0.2% galactose) in particular is limited due to rather wide flux confidence intervals resulting from the ^13^C FVA (García Martín et al., 2015). The best fit values, nonetheless, are consistent with these trends.

### 3.7 Aspartate/Threonine Biosynthesis

**Figure 6:**
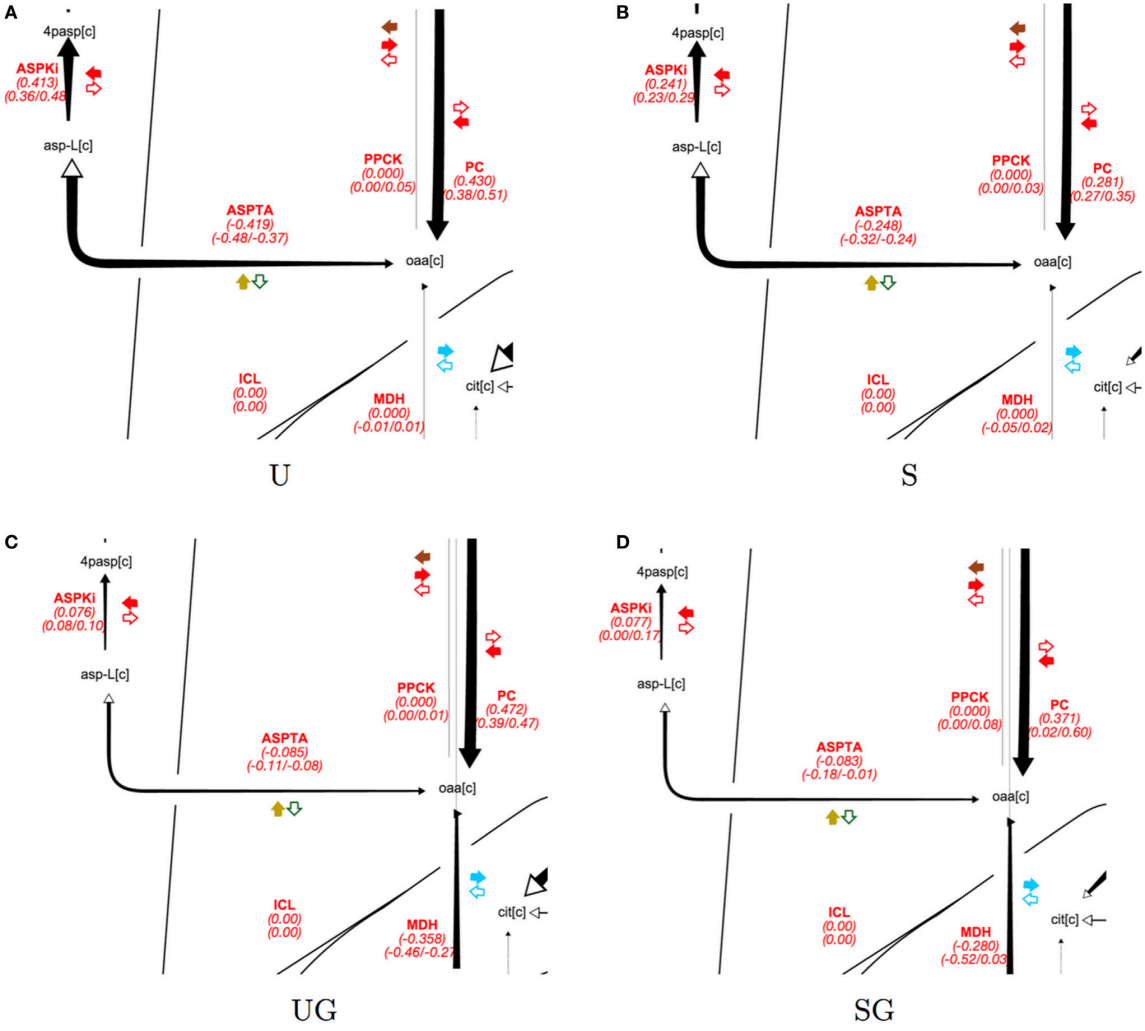
**Split of pyruvate carboxylase flux split for all strain/condition pairs**. Strain/condition pair designations U, S, UG, and SG refer to base in 2% glucose (**A**), sip1Δ in 2% glucose (**B**), base in 2% glucose + 0.2% galactose (**C**), and sip1Δ in 2% glucose + 0.2% galactose (**D**), respectively. Flux values and their absolute ranges obtained from the ELVA are presented in Table 5. Pyruvate carboxylase flux splits between aspartic acid biosynthesis and generation of cytosolic malate. Deletion of *SIP1* in glucose-repressing conditions (U vs S and UG vs SG) resulted in decreased pyruvate carboxylase (PC) and aspartate/threonine biosynthesis (ASPTA). Adding galactose to the medium decreased aspartate/threonine biosynthesis for both U and S. See previous figure for diagram explanation. For a description of colorful cofactor arrows, see the Fits and ELVA plots subsection in the Results section. Flux maps for the full model can be found in the Supplementary Material.

In the absence of galactose, both strains appear to direct pyruvate flux mainly toward ethanol and aspartate/threonine biosynthesis. The split of pyruvate carboxylase flux toward aspartate/threonine biosynthesis and production of cytosolic malate is displayed in Figure 6 for all strain/condition pairs and individual flux values and their absolute ranges from the ELVA are presented in Table 5. Deletion of *SIP1* in glucose-repressing conditions (base in 2% glucose vs sip1Δ in 2% glucose) resulted in a ~35% decrease in pyruvate carboxylase and a ~41% decrease in flux toward aspartate/threonine biosynthesis. The presence of 0.2% galactose in the medium of the base strain (base in 2% glucose vs base in 2% glucose + 0.2% galactose) resulted in a ~10% increase in pyruvate carboxylase activity and a ~80% decrease in flux toward aspartate/threonine biosynthesis. Similarly, adding galactose to the medium of the *sip1*Δ mutant (sip1Δ in 2% glucose vs sip1Δ in 2% glucose + 0.2% galactose) resulted in a ~32% increase in pyruvate carboxylase flux and a ~66% decrease in flow toward aspartate/threonine biosynthesis. As before, the flux confidence intervals for SG are quite wide, limiting the quality of the inferences for this particular strain/condition.

## 4 Discussion

We previously (Shymansky, 2011) observed an increase in maximum specific growth rate upon deletion of *SIP1* in medium containing both glucose and galactose in a background similar to this study (S288c *ura3*Δ *gal1*Δ). This increase was unreported in the literature and, thus, attracted our attention for further investigation. We chose to perform an exploratory analysis of this unreported phenotype from a fluxomic perspective in a similar set of base and *sip1*Δ mutant strains constructed in a CEN.PK113-7D *ura3*Δ *gal1*Δ background. Exponential-phase intracellular flux profiles were inferred from ^13^C tracer experiments using 2S-^13^C MFA for all four strain/condition pairs and compared. Our hope was to compare the redistribution of fluxes, if any, resulting from deletion of *SIP1* and/or inclusion of galactose in glucose medium and identify any resulting patterns.

A number of unexpected phenotypic differences were encountered during these investigations. Glucose repression appeared to be lessened at the 1/10 galactose-to-glucose ratio used. Under glucose-repressing conditions, the cell is expected to ignore other substrates. However, here, we find that the presence of galactose for the base strain results in an unpublished increase in growth rate (Table 4; Figure 3) and redistribution of flux from the PPP to the mitochondria and subsequent valine production. More specifically, this additional sugar resulted in decreased flux through the PPP and increased flow through mitochondrial pyruvate, via import of pyruvate and NADP-dependent malic enzyme, with subsequent cytosolic production of valine (see Figures S9–S12 in Supplementary Material). Additionally, switching the *sip1*Δ mutant from glucose-only to mixed glucose/galactose medium resulted in a similar increase in maximum specific growth rate and decrease in PPP flux.

The most striking implication of these results is that glucose repression in mixed glucose/galactose medium is not as strict as we anticipated, at least not in a *gal*Δ background. We find this apparent violation of glucose repression plausible based on a recent reevaluation of this phenomenon. There are instances in the literature of galactose regulation (Escalante-Chong et al., 2015; Venturelli et al., 2015; Wang et al., 2015), activating while glucose is being actively consumed, even without the loss of galactose metabolism via knockout of *GAL1*. Escalante-Chong et al. (2015) demonstrated the existence of a ratio-sensing mechanism using, among other efforts, a series of microwell experiments where they monitored the expression of yellow fluorescent protein (YFP) under a *GAL1* promoter over a range of galactose-to-glucose ratios in an S288c background. They determined that the range of galactose-to-glucose ratios was barely explored in the literature and that YFP was expressed past a particular galactose/glucose concentration ratio. The beginning of this expression activation happened to occur at the same 1/10 ratio of galactose-to-glucose used in this study. Although their strain background was S288c (compared to CEN.PK113-7D in this study) and they monitored growth in microwell plates (instead of shake flasks), it is possible that a similar effect might be occurring, even if CEN.PK113-7D is known to exhibit phenotypic differences relative to S288c (Nijkamp et al., 2012). More specifically, it is possible that galactose is entering the cell due to this ratio-sensing mechanism and indirectly influencing the growth rate. The galactose cannot contribute material directly to cellular mass nor to that flowing through the metabolic network due to the *GAL1* knockout. We speculate that the accompanying flux redistribution represents some sort of sensing of and preparation for degradation of galactose, a phenomenon that has been previously reported for *S. cerevisiae* (New et al., 2014; Venturelli et al., 2015; Wang et al., 2015).

We found deletion of *SIP1* to have important effects on glucose-repressed metabolism. *SIP1* appears to be an obligatory footnote in yeast glucose repression literature. If mentioned at all, it is mostly described as the *β*-subunit of the Snf1 kinase complex. Sometimes details about its localization or role in sequestering the complex in the vacuole are mentioned but it appears to be largely ignored. We suspect this is due to the lack of a growth phenotype accompanying knockout of *SIP1*. To our knowledge, this and our previous work (Shymansky, 2011) are the only studies to even attempt to infer fluxes in a *SIP1* null mutant and to report extracellular exchange rates other than for glucose. We were surprised to find that, despite no difference in growth rate between both strains, deletion of *SIP1* in glucose-only medium appeared to effectively decrease absolute extracellular ethanol and glucose exchange rates and decrease flow toward aspartate and threonine biosynthesis. The same trends were either not observed in mixed glucose/galactose medium or the confidence intervals of these patterns were too wide to definitively note differences. These differences resulting from deletion of *SIP1* are in contrast to a previous study (Zhang et al., 2010) that noted no differences in ethanol yield, growth rate, nor glucose exchange rate upon knockout of *SIP1*. However, both strains in our study were Gal1^−^ while the 2010 Zhang *et al*. strains had intact *GAL1* genes.

Our results are consistent with our mechanistic understanding that Sip1 is a negative regulator of the *GAL* system. Deletion of *SIP1* appears to amplify the effect galactose has on growth rate. Why deletion of *SIP1* would decrease glucose consumption, ethanol excretion, and aspartate/threonine biosynthesis rates is unclear, though it appears the cell is diverting additional resources toward maintaining its growth rate. Additionally, it appears that galactose needed to be present to see an increase in maximum specific growth rate from deletion of *SIP1*. Aside from the differences noted above, normal patterns of glucose repression (e.g., ethanol fermentation and repression of TCA and glyoxylate cycle activity) appeared in all four strain/condition pairs. Unfortunately, our flux confidence intervals are too wide to meaningfully compare the flux profile of the *sip1*Δ mutant, other than the PPP patterns, in mixed glucose/galactose medium with the remaining three strain/condition pairs. Thus, our analysis regarding it is limited to its higher growth rate and this section of the network. While this may seem like a disadvantage, it highlights a strength of our analysis. The combination of the ELVA and ^13^C FVA allows us to judge the consistency of our model, data, inferred flux profiles, and simulated labeling and when it is or is not appropriate to derive further conclusions.

In this study, we have shown how to go from gross phenotypic changes (e.g., growth rate, glucose, and ethanol input changes) to mechanistic metabolic insights by using modeling techniques based on constraining comprehensive genome-scale models by ^13^C labeling data. In particular, the use of the 2-scale version of ^13^C MFA, notably expanding the core set of reactions until acceptable simulated labeling ranges were obtained, led to our insights in mitochondrial transport. Most ^13^C MFA studies do not include these mitochondrial transport reactions. In fact, the initial carbon transition model in this study did not include them. It was only through the process of adding them to tighten the computational error in the ELVA plots and then visualizing the fluxes that it became apparent that this cycle was occurring.

To our knowledge, this is the first published study to investigate the relative effects of the presence of galactose and knockout of *SIP1* in normally carbon repressing conditions from a fluxomic perspective. We also encountered increases in growth rate when galactose was present in normally glucose-repressing medium not found in the scientific literature. It is also one of the first to apply 2S-^13^C MFA to model yeast. This model (as every modeling endeavor) needs to rely on a variety of assumptions (e.g., steady state conditions, completeness of the genome-scale stoichiometry, cell homogeneity, lack of flux flow from metabolic periphery to core reactions, no accumulation of intermediate metabolites, etc.). Some of the assumptions the model is based on may fail, so it is advisable that these insights be confirmed through further experiments (e.g., labeling measurements for additional metabolites or proteomics/transcriptomics studies). However, the model is able to take a profusion of disconnected quantitative data (e.g., growth rate changes, ethanol and acetate excretion rates, labeling patterns) and convert them into insights of what types of metabolic changes the *SIP1* knockout (or the presence of galactose) are likely to produce in the cell for further interrogation, similarly to what has been demonstrated before in terms of biofuel production increases (Ghosh et al., 2016).

## Author Contributions

CS conceived of the project, did the experiments, analyzed the data, and wrote the paper. GW and EB produced the metabolomics data and helped write the paper. JG helped performed experiments and wrote the paper. AA and AM helped write the paper. HM conceived of the project and helped analyze the data and write the paper. JK conceived of the project and helped write the paper.

## Conflict of Interest Statement

The authors declare that the research was conducted in the absence of any commercial or financial relationships that could be construed as a potential conflict of interest.

## Supplementary Material

Additional file 1 **Table S1**. Media and component concentrations. **Table S2**. Intracellular metabolite labeling distribution input for base in 2% glucose. **Table S3**. Intracellular metabolite labeling distribution SD input for base in 2% glucose. **Table S4**. Intracellular metabolite labeling distribution input for sip1Δ in 2% glucose. **Table S5**. Intracellular metabolite labeling distribution SD input for sip1Δ in 2% glucose. **Table S6**. Intracellular metabolite labeling distribution input for base in 2% glucose + 0.2% galactose. **Table S7**. Intracellular metabolite labeling distribution SD input for base in 2% glucose + 0.2% galactose. **Table S8**. Intracellular metabolite labeling distribution input for sip1Δ in 2% glucose + 0.2% galactose. **Table S9**. Intracellular metabolite labeling distribution SD input for sip1Δ in 2% glucose + 0.2% galactose. **Figure S1**. Detailed measured/simulated MDV fits for sip1Δ in 2% glucose. **Figure S2**. Detailed measured/simulated MDV fits for base in 2% glucose + 0.2% galactose. **Figure S3**. Detailed measured/simulated MDV fits for sip1Δ in 2% glucose + 0.2% galactose. **Figure S4**. ELVA plots for all four strain/condition pairs. **Figure S5**. Flux profile corresponding to base in 2% glucose. **Figure S6**. Flux profile corresponding to sip1Δ in 2% glucose. **Figure S7**. Flux profile corresponding to base in 2% glucose + 0.2% galactose. **Figure S8**. Flux profile corresponding to sip1Δ in 2% glucose + 0.2% galactose. **Figure S9**. Zoomed in section of flux map corresponding to mitochondrial import of pyruvate/malate and production of branched-chain amino acids for base in 2% glucose. **Figure S10**. Zoomed in section of flux map corresponding to mitochondrial import of pyruvate/malate and production of branched-chain amino acids for sip1Δ in 2% glucose. **Figure S11**. Zoomed in section of flux map corresponding to mitochondrial import of pyruvate/malate and production of branched-chain amino acids for base in 2% glucose + 0.2% galactose. **Figure S12**. Zoomed in section of flux map corresponding to mitochondrial import of pyruvate/malate and production of branched-chain amino acids for sip1Δ in 2% glucose + 0.2% galactose.

Additional file 2 **Jupyter notebook QMM library 2S-^13^C MFA calculation code and input files**. Zip file containing a Jupyter notebook file (two_scale_sip1_calculations.ipynb) used to run all flux calculations, another Jupyter notebook (extracellular_flux_calculation_example.ipynb) demonstrating the an example extracellular flux calculation, the QMM library code necessary to infer flux profiles via 2S-^13^C MFA, text file inputs for the glucose feed labeling, extracellular fluxes, measured intracellular metabolite MDVs, the standard deviations corresponding to these measured MDVS, genome-scale model, and the core reaction network files corresponding to strain/condition pairs U, S, UG, and SG.

Additional file 3 **Example extracellular flux calculation and derivation of formula**. This is the.html file corresponding to the Jupyter notebook found in Additional file 2 that demonstrates the derivation of the formula used to calculate extracellular fluxes and an example of its use.
