# Supplementary material for: Flux-Enabled Exploration of the Role of Sip1 in Galactose Yeast Metabolism
Source: Front Bioeng Biotechnol. 2017 May 24;5:31. doi: 10.3389/fbioe.2017.00031 (PMC5443151; doi:10.3389/fbioe.2017.00031)
Supplement: Additional file 1 — Table S1. Media and component concentrations. Table S2. Intracellular metabolite labeling distribution input for base in 2% glucose. Table S3. Intracellular metabolite labeling distribution SD input for base in 2% glucose. Table S4. Intracellular metabolite labeling distribution input for sip1Δ in 2% glucose. Table S5. Intracellular metabolite labeling distribution SD input for sip1Δ in 2% glucose. Table S6. Intracellular metabolite labeling distribution input for base in 2% glucose + 0.2% galactose. Table S7. Intracellular metabolite labeling distribution SD input for base in 2% glucose + 0.2% galactose. Table S8. Intracellular metabolite labeling distribution input for sip1Δ in 2% glucose + 0.2% galactose. Table S9. Intracellular metabolite labeling distribution SD input for sip1Δ in 2% glucose + 0.2% galactose. Figure S1. Detailed measured/simulated MDV fits for sip1Δ in 2% glucose. Figure S2. Detailed measured/simulated MDV fits for base in 2% glucose + 0.2% galactose. Figure S3. Detailed measured/simulated MDV fits for sip1Δ in 2% glucose + 0.2% galactose. Figure S4. ELVA plots for all four strain/condition pairs. Figure S5. Flux profile corresponding to base in 2% glucose. Figure S6. Flux profile corresponding to sip1Δ in 2% glucose. Figure S7. Flux profile corresponding to base in 2% glucose + 0.2% galactose. Figure S8. Flux profile corresponding to sip1Δ in 2% glucose + 0.2% galactose. Figure S9. Zoomed in section of flux map corresponding to mitochondrial import of pyruvate/malate and production of branched-chain amino acids for base in 2% glucose. Figure S10. Zoomed in section of flux map corresponding to mitochondrial import of pyruvate/malate and production of branched-chain amino acids for sip1Δ in 2% glucose. Figure S11. Zoomed in section of flux map corresponding to mitochondrial import of pyruvate/malate and production of branched-chain amino acids for base in 2% glucose + 0.2% galactose. Figure S12. Zoomed in section of flux map corresponding to mit [file Data_Sheet_1.PDF]

Supplementary Material:
Flux-enabled exploration of the role of Sip1 in galactose yeast metabolism

Christopher M. Shymansky [1,2,3], George Wang [1,2], Edward E.K. Baidoo [1,2], Jennifer Gin [1,2], Amanda Reider Apel [1,2], Aindrila Mukhopadhyay [1,2], Héctor García Martín [1,2*], and Jay D. Keasling [1,2,3,4,5]

*Correspondence:
Héctor García Martín
hgmartin@lbl.gov

1 SUPPLEMENTARY TABLES AND FIGURES

1.1 Tables

Table S1. Media and component concentrations

Media	Component	Concentration [g/100 mL]
Min	Glucose	2
	Yeast nitrogen base w/o amino acids	0.67
Min+Gal	Glucose	2
	Galactose	0.2
	Yeast nitrogen base w/o amino acids	0.67
Min+Ura+5-FOA	Glucose	2
	Yeast nitrogen base w/o amino acids	0.67
	Uracil (Ura)	0.002
	5-Fluoroorotic acid (5-FOA)	0.1
YPD	Glucose	2
	Bacto-yeast extract	1
	Bacto-peptone	2
YPD+G418	Glucose	2
	Bacto-yeast extract	1
	Bacto-peptone	2
	Geneticin (G418)	0.02
Sc-Ura	Glucose	2
	Yeast nitrogen base w/o amino acids	0.67
	CSM-Ura	0.077
pSH47	Glucose	0.2
	Galactose	1.8
	Yeast nitrogen base w/o amino acids	0.67
	CSM-Ura	0.077

Note: All plates contained 2 g/100 mL bacto-agar

Table S2. Intracellular metabolite labeling distribution input for base in 2% glucose

```
m0 m1 m2 m3 m4 m5 m6 m7 m8 m9
3pg M-0 0.3936 0.3963 0.0238 0.1863
Ala M-0 0.3854 0.4034 0.0398 0.1713
Arg M-0 0.1045 0.2643 0.3117 0.2089 0.0864 0.0217 0.0026
Asp M-0 0.3176 0.3810 0.1122 0.1429 0.0464
Gln M-0 0.1352 0.2860 0.3219 0.1935 0.0634 0.0000
Glu M-0 0.1219 0.2855 0.3123 0.2021 0.0655 0.0127
Ile M-0 0.1466 0.2877 0.2536 0.1739 0.0954 0.0427 0.0000
Leu M-0 0.0705 0.2265 0.3238 0.1862 0.1434 0.0496 0.0000
Lys M-0 0.0662 0.1883 0.2944 0.2476 0.1359 0.0597 0.0080
Phe M-0 0.0979 0.2272 0.2021 0.1656 0.1207 0.0898 0.0533 0.0150 0.0127
0.0158
Thr M-0 0.3247 0.3787 0.1133 0.1531 0.0302
Tyr M-0 0.1012 0.2292 0.1996 0.1724 0.1220 0.0900 0.0563 0.0000 0.0293
0.0000
Val M-0 0.1727 0.3367 0.2583 0.1684 0.0267 0.0371
fdp M-0 0.1288 0.3830 0.1744 0.1164 0.1365 0.0119 0.0489
cit_m M-0 0.1279 0.2737 0.2643 0.1841 0.0993 0.0422 0.0085
succ_m M-0 0.2665 0.3554 0.1848 0.1554 0.0379
```

Table S3. Intracellular metabolite labeling distribution standard deviation input for base in 2% glucose

```
m0 m1 m2 m3 m4 m5 m6 m7 m8 m9
3pg M-0 0.0059 0.0053 0.0020 0.0047
Ala M-0 0.0040 0.0025 0.0013 0.0005
Arg M-0 0.0023 0.0088 0.0046 0.0025 0.0007 0.0003 0.0000
Asp M-0 0.0082 0.0063 0.0130 0.0054 0.0041
Gln M-0 0.0017 0.0069 0.0025 0.0009 0.0032 0.0000
Glu M-0 0.0077 0.0091 0.0083 0.0053 0.0019 0.0010
Ile M-0 0.0076 0.0066 0.0045 0.0057 0.0032 0.0015 0.0000
Leu M-0 0.0047 0.0323 0.0396 0.1083 0.0219 0.0117 0.0000
Lys M-0 0.0005 0.0025 0.0030 0.0040 0.0011 0.0094 0.0007
Phe M-0 0.0034 0.0092 0.0047 0.0046 0.0033 0.0020 0.0029 0.0150 0.0009
0.0071
Thr M-0 0.0070 0.0083 0.0030 0.0021 0.0198
Tyr M-0 0.0018 0.0085 0.0017 0.0027 0.0026 0.0014 0.0019 0.0033 0.0033
0.0033
Val M-0 0.0090 0.0171 0.0108 0.0097 0.0463 0.0027
fdp M-0 0.0032 0.0090 0.0023 0.0156 0.0049 0.0002 0.0058
cit_m M-0 0.0026 0.0037 0.0004 0.0005 0.0027 0.0007 0.0004
succ_m M-0 0.0159 0.0053 0.0204 0.0015 0.0008
```

Table S4. Intracellular metabolite labeling distribution input for sip1Δ in 2% glucose

```
m0 m1 m2 m3 m4 m5 m6 m7 m8 m9
3pg M-0 0.3895 0.3977 0.0258 0.1870
Ala M-0 0.3879 0.3976 0.0405 0.1740
Arg M-0 0.1063 0.2631 0.3121 0.2083 0.0866 0.0210 0.0026
Asp M-0 0.3328 0.3948 0.0780 0.1485 0.0460
Gln M-0 0.1341 0.2882 0.3199 0.1895 0.0655 0.0028
Glu M-0 0.1209 0.2922 0.3090 0.2011 0.0646 0.0122
Ile M-0 0.1524 0.2956 0.2512 0.1680 0.0902 0.0425 0.0000
Leu M-0 0.0684 0.2018 0.2916 0.2578 0.1370 0.0435 0.0000
Lys M-0 0.0680 0.1893 0.2972 0.2526 0.1391 0.0466 0.0071
Phe M-0 0.0972 0.2358 0.2087 0.1565 0.1181 0.0883 0.0518 0.0213 0.0126
0.0098
Thr M-0 0.3160 0.3789 0.1106 0.1490 0.0456
Tyr M-0 0.1019 0.2377 0.1967 0.1728 0.1223 0.0920 0.0556 0.0000 0.0171
0.0039
Val M-0 0.1759 0.3450 0.2679 0.1728 0.0000 0.0384
fdp M-0 0.1239 0.3716 0.1749 0.1313 0.1445 0.0128 0.0410
cit_m M-0 0.1269 0.2876 0.2476 0.1894 0.0992 0.0420 0.0072
succ_m M-0 0.2687 0.3553 0.1809 0.1562 0.0388
```

Table S5. Intracellular metabolite labeling distribution standard deviation input for sip1Δ in 2% glucose

```
m0 m1 m2 m3 m4 m5 m6 m7 m8 m9
3pg M-0 0.0140 0.0164 0.0025 0.0046
Ala M-0 0.0021 0.0035 0.0002 0.0031
Arg M-0 0.0035 0.0063 0.0071 0.0087 0.0009 0.0006 0.0001
Asp M-0 0.0054 0.0025 0.0006 0.0047 0.0046
Gln M-0 0.0054 0.0284 0.0129 0.0073 0.0029 0.0000
Glu M-0 0.0061 0.0058 0.0019 0.0005 0.0022 0.0002
Ile M-0 0.0134 0.0035 0.0046 0.0049 0.0018 0.0003 0.0000
Leu M-0 0.0032 0.0061 0.0099 0.0020 0.0016 0.0031 0.0000
Lys M-0 0.0009 0.0024 0.0012 0.0018 0.0017 0.0015 0.0003
Phe M-0 0.0027 0.0037 0.0026 0.0008 0.0022 0.0010 0.0123 0.0007 0.0002
0.0010
Thr M-0 0.0057 0.0029 0.0039 0.0010 0.0009
Tyr M-0 0.0024 0.0037 0.0066 0.0018 0.0013 0.0007 0.0010 0.0000 0.0083
0.0039
Val M-0 0.0050 0.0015 0.0019 0.0030 0.0000 0.0007
fdp M-0 0.0030 0.0127 0.0037 0.0156 0.0024 0.0001 0.0008
cit_m M-0 0.0096 0.0103 0.0125 0.0074 0.0044 0.0033 0.0005
succ_m M-0 0.0078 0.0049 0.0128 0.0006 0.0005
```

Table S6. Intracellular metabolite labeling distribution input for base in 2% glucose + 0.2% galactose

```
m0 m1 m2 m3 m4 m5 m6 m7 m8 m9
3pg M-0 0.3833 0.4029 0.0239 0.1898
Ala M-0 0.3905 0.3920 0.0407 0.1768
Arg M-0 0.1058 0.2587 0.3098 0.2134 0.0883 0.0213 0.0026
Asp M-0 0.3278 0.3820 0.1051 0.1435 0.0415
Gln M-0 0.1345 0.2884 0.3160 0.1879 0.0609 0.0122
Glu M-0 0.1248 0.2953 0.3100 0.1944 0.0644 0.0110
Ile M-0 0.1753 0.2909 0.2398 0.1615 0.0842 0.0406 0.0077
Leu M-0 0.0756 0.2009 0.2896 0.2496 0.1318 0.0440 0.0084
Lys M-0 0.0674 0.1910 0.3000 0.2515 0.1372 0.0456 0.0073
Phe M-0 0.0991 0.2250 0.2263 0.1539 0.1169 0.0894 0.0456 0.0266 0.0113
0.0059
Thr M-0 0.3213 0.3835 0.1072 0.1459 0.0421
Tyr M-0 0.0973 0.2133 0.2118 0.1540 0.1237 0.0939 0.0515 0.0000 0.0264
0.0281
Val M-0 0.1582 0.3213 0.2497 0.1577 0.0807 0.0324
fdp M-0 0.1411 0.3165 0.1850 0.1696 0.1283 0.0119 0.0476
cit_m M-0 0.1279 0.2726 0.2662 0.1801 0.1058 0.0403 0.0073
succ_m M-0 0.2636 0.3593 0.1876 0.1531 0.0364
```

Table S7. Intracellular metabolite labeling distribution standard deviation input for base in 2% glucose + 0.2% galactose

```
m0 m1 m2 m3 m4 m5 m6 m7 m8 m9
3pg M-0 0.0078 0.0083 0.0011 0.0127
Ala M-0 0.0026 0.0016 0.0019 0.0011
Arg M-0 0.0013 0.0013 0.0013 0.0013 0.0012 0.0003 0.0000
Asp M-0 0.0112 0.0054 0.0036 0.0030 0.0017
Gln M-0 0.0012 0.0028 0.0005 0.0021 0.0004 0.0007
Glu M-0 0.0009 0.0013 0.0003 0.0012 0.0004 0.0008
Ile M-0 0.0067 0.0038 0.0024 0.0020 0.0008 0.0010 0.0002
Leu M-0 0.0019 0.0042 0.0029 0.0025 0.0009 0.0010 0.0009
Lys M-0 0.0008 0.0014 0.0012 0.0007 0.0021 0.0010 0.0003
Phe M-0 0.0058 0.0082 0.0046 0.0110 0.0042 0.0040 0.0020 0.0003 0.0004
0.0002
Thr M-0 0.0017 0.0021 0.0023 0.0022 0.0019
Tyr M-0 0.0015 0.0105 0.0076 0.0021 0.0017 0.0025 0.0010 0.0000 0.0028
0.0022
Val M-0 0.0012 0.0021 0.0018 0.0007 0.0031 0.0003
fdp M-0 0.0102 0.0208 0.0196 0.0036 0.0015 0.0003 0.0025
cit_m M-0 0.0015 0.0051 0.0045 0.0026 0.0019 0.0012 0.0003
succ_m M-0 0.0108 0.0046 0.0146 0.0015 0.0007
```

Table S8. Intracellular metabolite labeling distribution input for sip1Δ in 2% glucose + 0.2% galactose

```
m0 m1 m2 m3 m4 m5 m6 m7 m8 m9
3pg M-0 0.3098 0.4402 0.0082 0.2419
Ala M-0 0.3950 0.3952 0.0386 0.1712
Arg M-0 0.1056 0.2602 0.3153 0.2094 0.0859 0.0211 0.0025
Asp M-0 0.3115 0.3878 0.1058 0.1506 0.0444
Gln M-0 0.1331 0.2794 0.3163 0.1864 0.0712 0.0135
Glu M-0 0.1201 0.2866 0.3043 0.2070 0.0707 0.0113
Ile M-0 0.1795 0.2859 0.2384 0.1637 0.0869 0.0405 0.0050
Leu M-0 0.1024 0.2124 0.2385 0.2606 0.1348 0.0464 0.0050
Lys M-0 0.0717 0.1888 0.2991 0.2506 0.1352 0.0473 0.0072
Phe M-0 0.1084 0.2161 0.2267 0.1552 0.1168 0.0882 0.0547 0.0209 0.0112
0.0018
Thr M-0 0.2808 0.3283 0.2193 0.1294 0.0423
Tyr M-0 0.1186 0.2301 0.1383 0.1833 0.1288 0.1009 0.0830 0.0000 0.0170
0.0000
Val M-0 0.1842 0.3021 0.2294 0.1528 0.0894 0.0421
fdp M-0 0.1068 0.4036 0.1500 0.1331 0.1220 0.0122 0.0723
cit_m M-0 0.1483 0.2825 0.2626 0.1938 0.0647 0.0431 0.0051
succ_m M-0 0.3319 0.3541 0.1146 0.1602 0.0392
```

Table S9. Intracellular metabolite labeling distribution standard deviation input for sip1Δ in 2% glucose + 0.2% galactose

```
m0 m1 m2 m3 m4 m5 m6 m7 m8 m9
3pg M-0 0.1021 0.0786 0.0115 0.0377
Ala M-0 0.0041 0.0034 0.0031 0.0033
Arg M-0 0.0006 0.0011 0.0016 0.0027 0.0002 0.0003 0.0000
Asp M-0 0.0058 0.0051 0.0040 0.0031 0.0007
Gln M-0 0.0024 0.0082 0.0063 0.0041 0.0119 0.0015
Glu M-0 0.0029 0.0088 0.0117 0.0180 0.0053 0.0011
Ile M-0 0.0146 0.0043 0.0057 0.0035 0.0013 0.0008 0.0035
Leu M-0 0.0319 0.0145 0.0725 0.0214 0.0052 0.0035 0.0037
Lys M-0 0.0047 0.0015 0.0024 0.0024 0.0024 0.0012 0.0004
Phe M-0 0.0117 0.0029 0.0087 0.0069 0.0044 0.0044 0.0071 0.0149 0.0004
0.0025
Thr M-0 0.0311 0.0452 0.0939 0.0161 0.0047
Tyr M-0 0.0208 0.0225 0.0981 0.0209 0.0133 0.0088 0.0316 0.0000 0.0240
0.0000
Val M-0 0.0445 0.0059 0.0112 0.0017 0.0634 0.0118
fdp M-0 0.0074 0.0198 0.0230 0.0059 0.0085 0.0006 0.0053
cit_m M-0 0.0252 0.0165 0.0122 0.0006 0.0458 0.0020 0.0023
succ_m M-0 0.1574 0.1669 0.0561 0.0755 0.0185
```

1.2 Figures

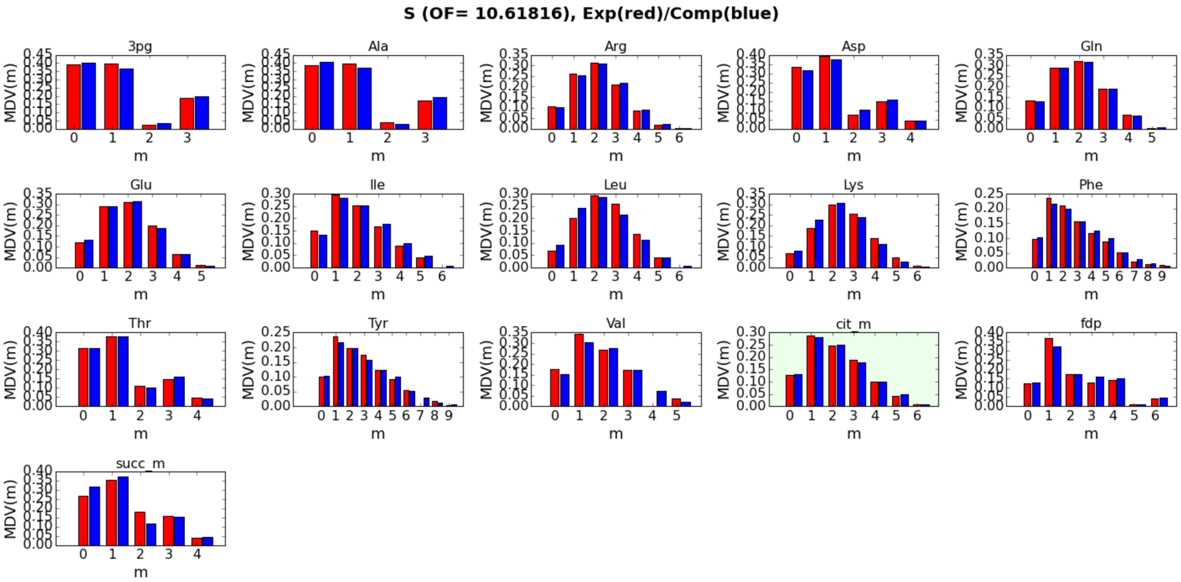

Figure S1. Detailed fits between simulated (blue bars) and measured (red bars) intracellular metabolite labeling distributions for sip1Δ in 2% glucose (S). The green box corresponds to a metabolite whose measured data was excluded from computations and the predicted labeling was compared to that measured

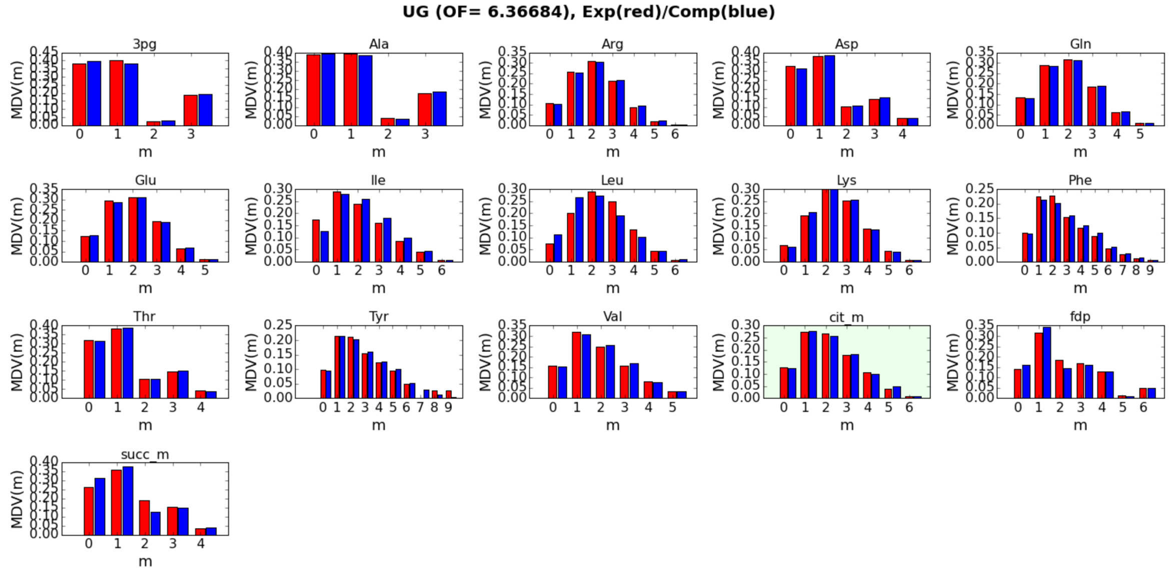

Figure S2. Detailed fits between simulated (blue bars) and measured (red bars) intracellular metabolite labeling distributions for base in 2% glucose + 0.2% galactose (UG). The green box corresponds to a metabolite whose measured data was excluded from computations and the predicted labeling was compared to that measured

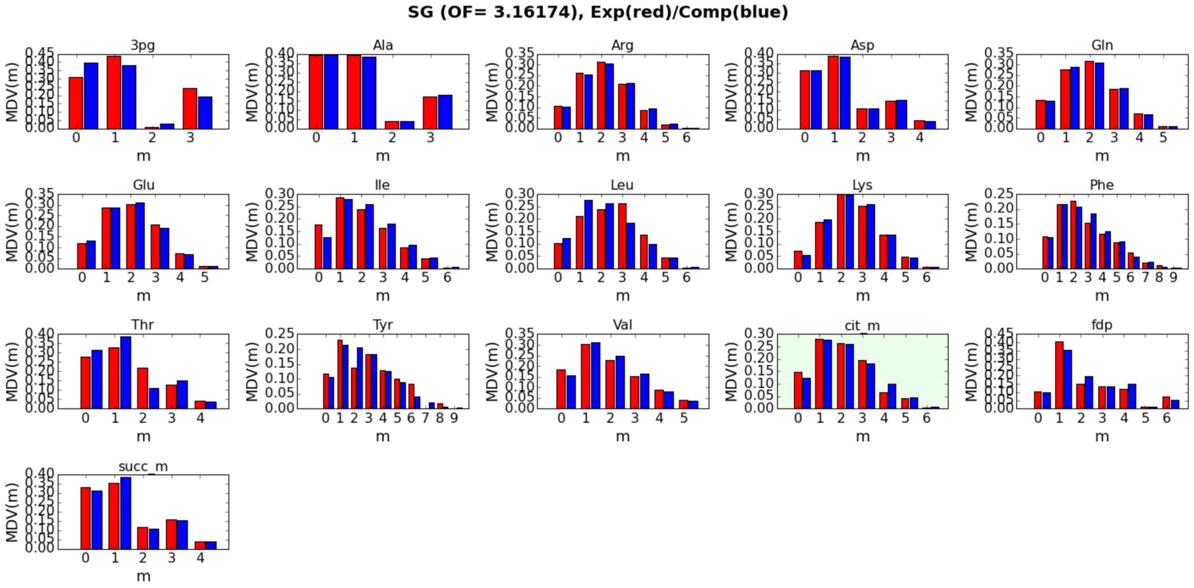

Figure S3. Detailed fits between simulated (blue bars) and measured (red bars) intracellular metabolite labeling distributions for sip1Δ in 2% glucose + 0.2% galactose (SG). The green box corresponds to a metabolite whose measured data was excluded from computations and the predicted labeling was compared to that measured

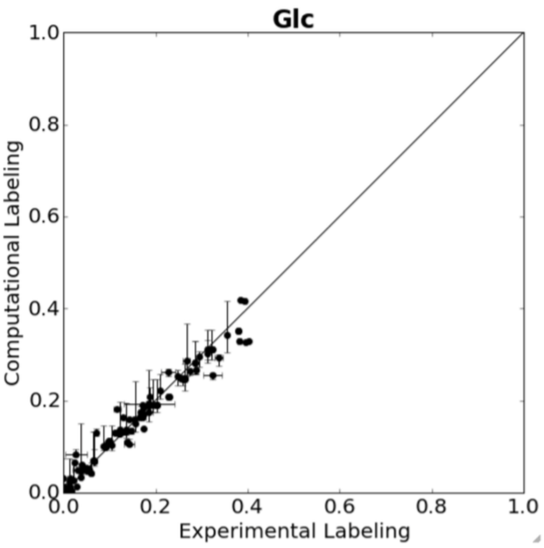

(a) U

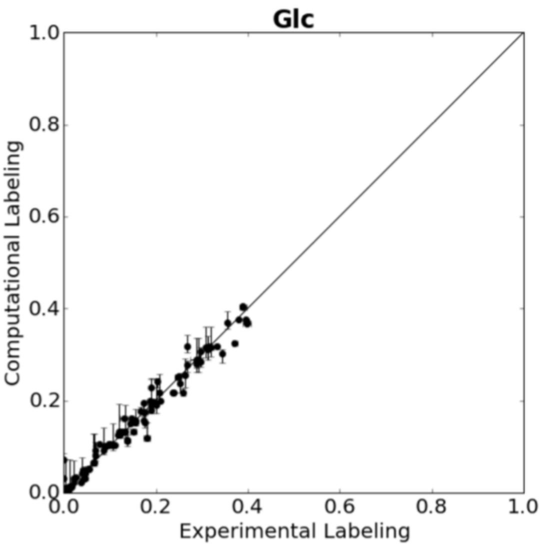

(b) S

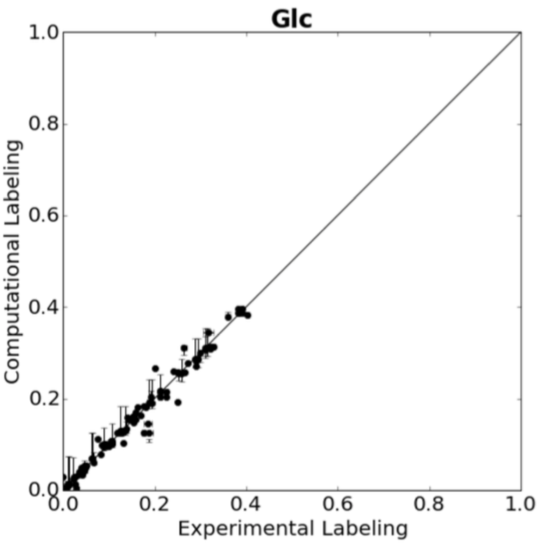

(c) UG

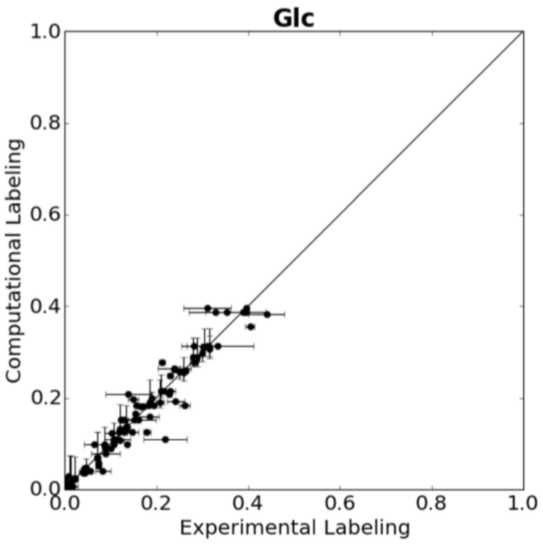

(d) SG

Figure S4. ELVA plots for all four strain/condition pairs. Strain/condition pair designations U, S, UG, and SG refer to base in 2% glucose, sip1Δ in 2% glucose, base in 2% glucose + 0.2% galactose, and sip1Δ in 2% glucose + 0.2% galactose, respectively. The x-axis represents the experimentally measured labeling values along with their errors. The y-axis represents the computationally predicted labeling values along with a ”computational error” that represents the maximum effect that non-core reactions (whose contribution to the carbon labeling is being ignored) could possibly have. Data lining along the diagonal represents an accurate description of experimental data by the model. Low computational errors indicate that the model is self-consistent and the non-core reactions can be safely disregarded when predicting labeling for the measured metabolites. This figure showcases the validity of the model.

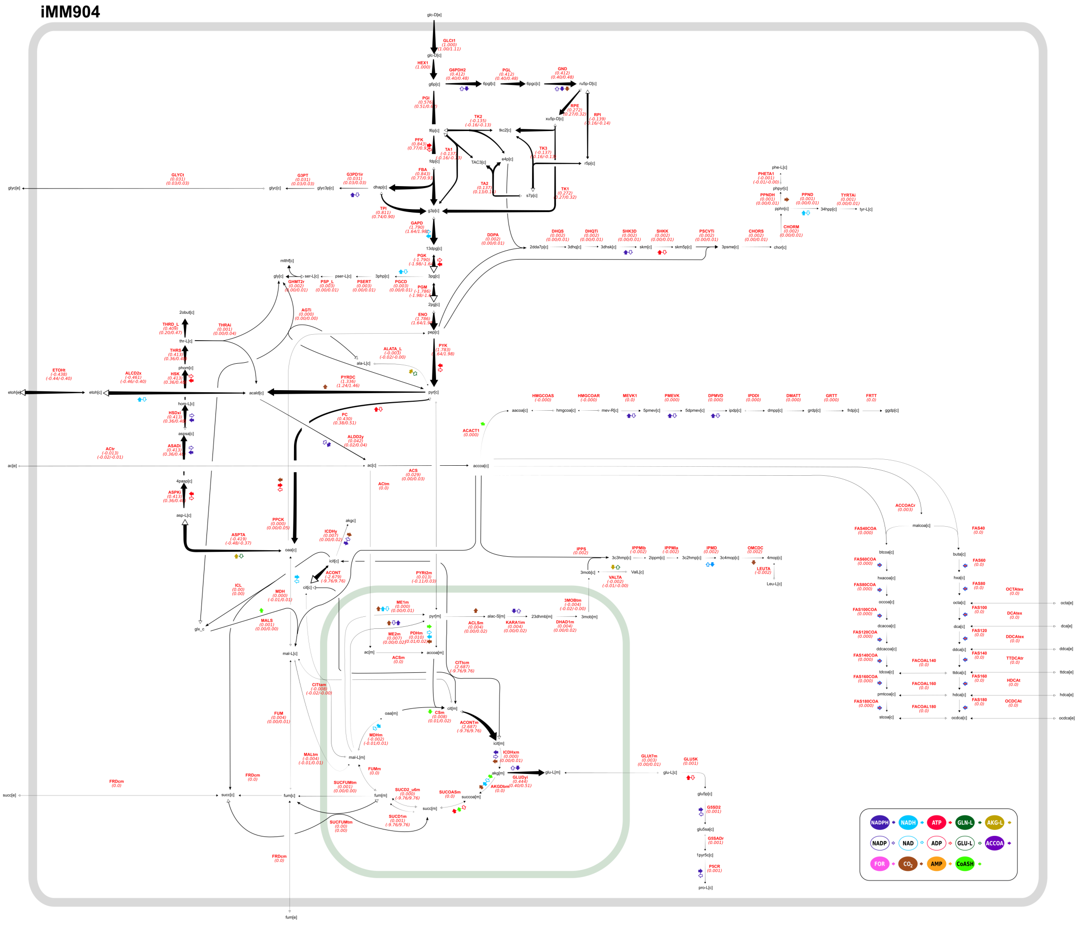

Figure S5. Whole flux profile for base in 2% glucose. For a description of colorful cofactor arrows see the Fits and ELVA plots subsection in the Results section.

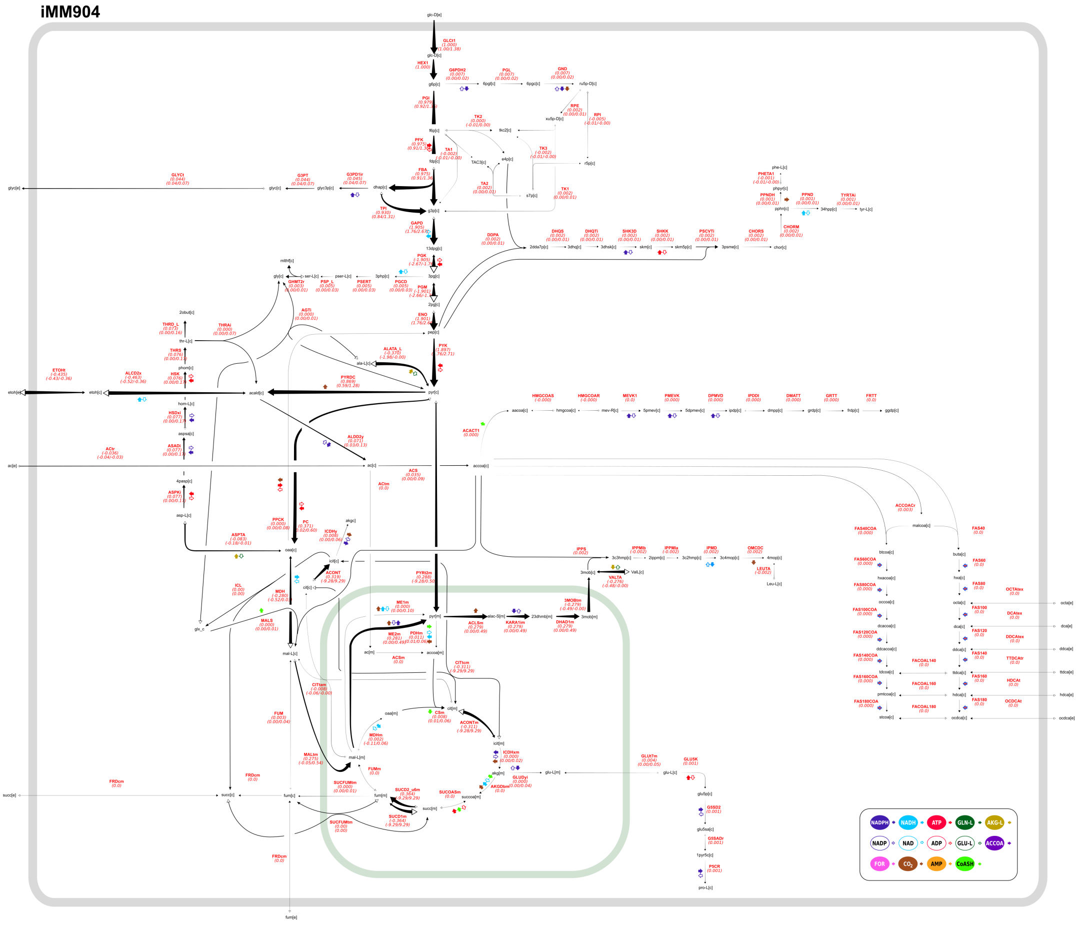

Figure S6. Whole flux profile for sip1Δ in 2% glucose. For a description of colorful cofactor arrows see the Fits and ELVA plots subsection in the Results section.

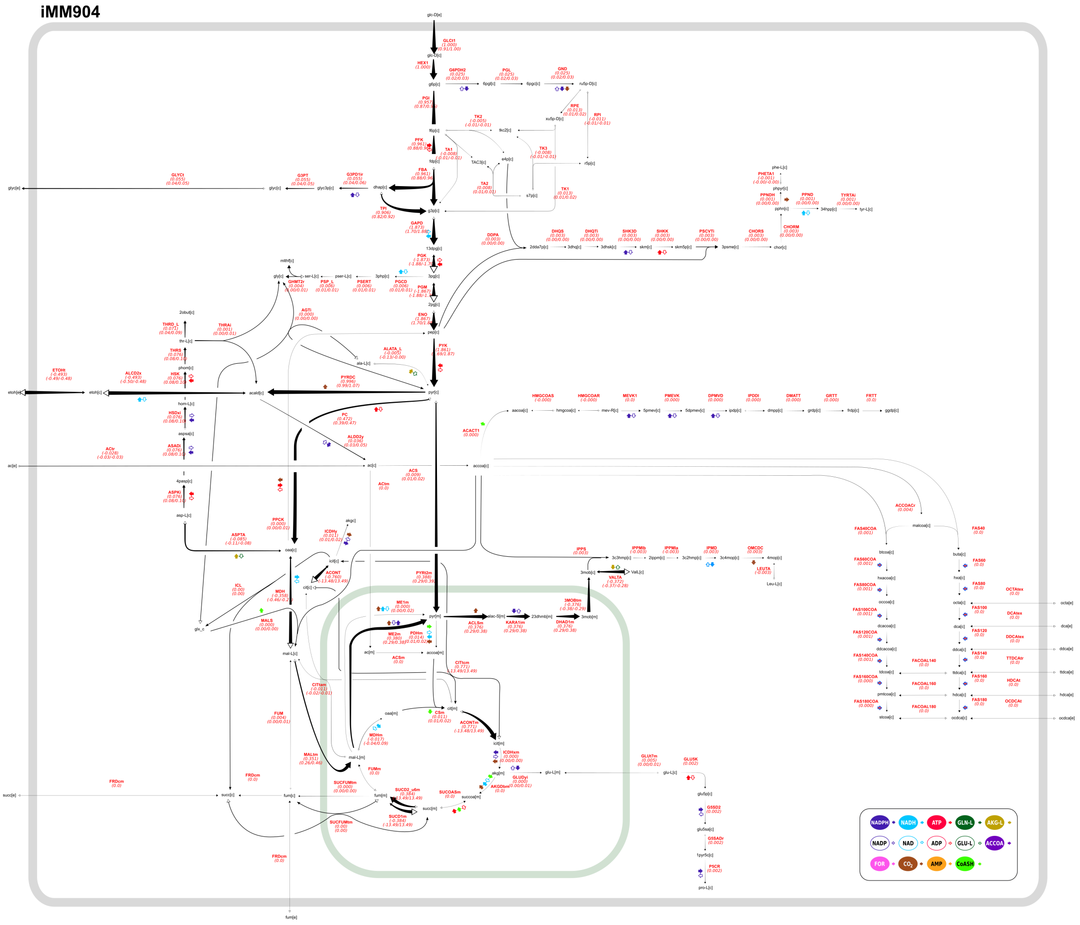

Figure S7. Whole flux profile for base in 2% glucose + 0.2% galactose. For a description of colorful cofactor arrows see the Fits and ELVA plots subsection in the Results section.

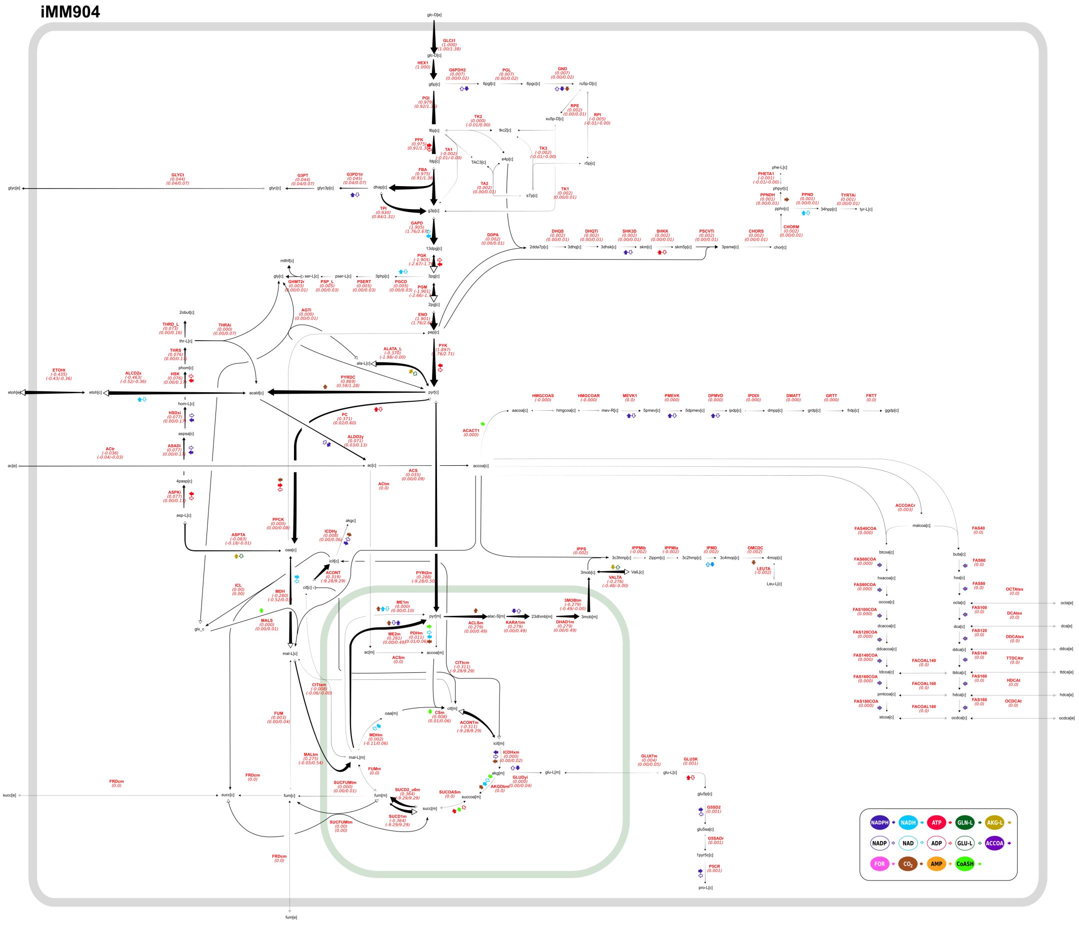

Figure S8. Whole flux profile for sip1Δ in 2% glucose + 0.2% galactose. For a description of colorful cofactor arrows see the Fits and ELVA plots subsection in the Results section.

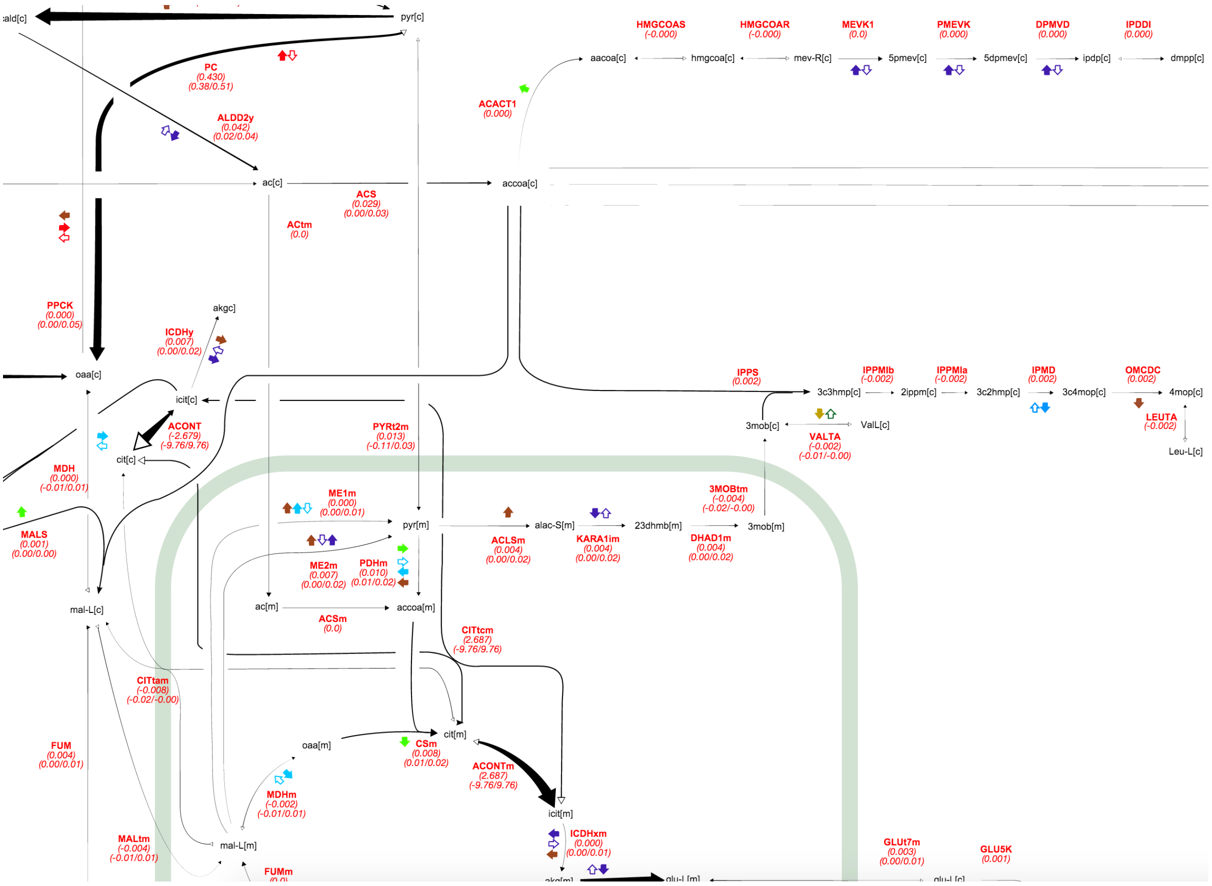

Figure S9. Mitochondrial import and branched-chain amino acid fluxes for base in 2% glucose. For a description of colorful cofactor arrows see the Fits and ELVA plots subsection in the Results section.

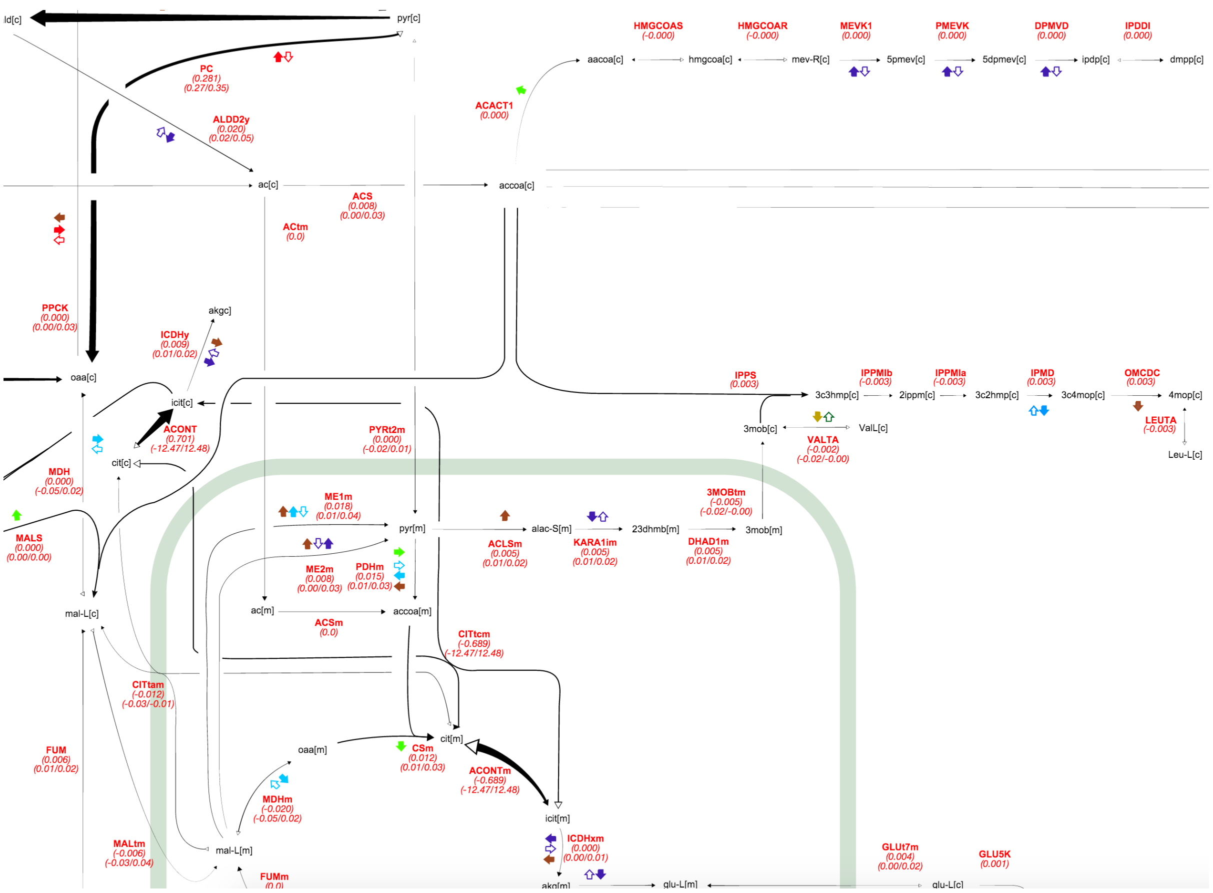

Figure S10. Mitochondrial import and branched-chain amino acid fluxes for sip1Δ in 2% glucose. For a description of colorful cofactor arrows see the Fits and ELVA plots subsection in the Results section.

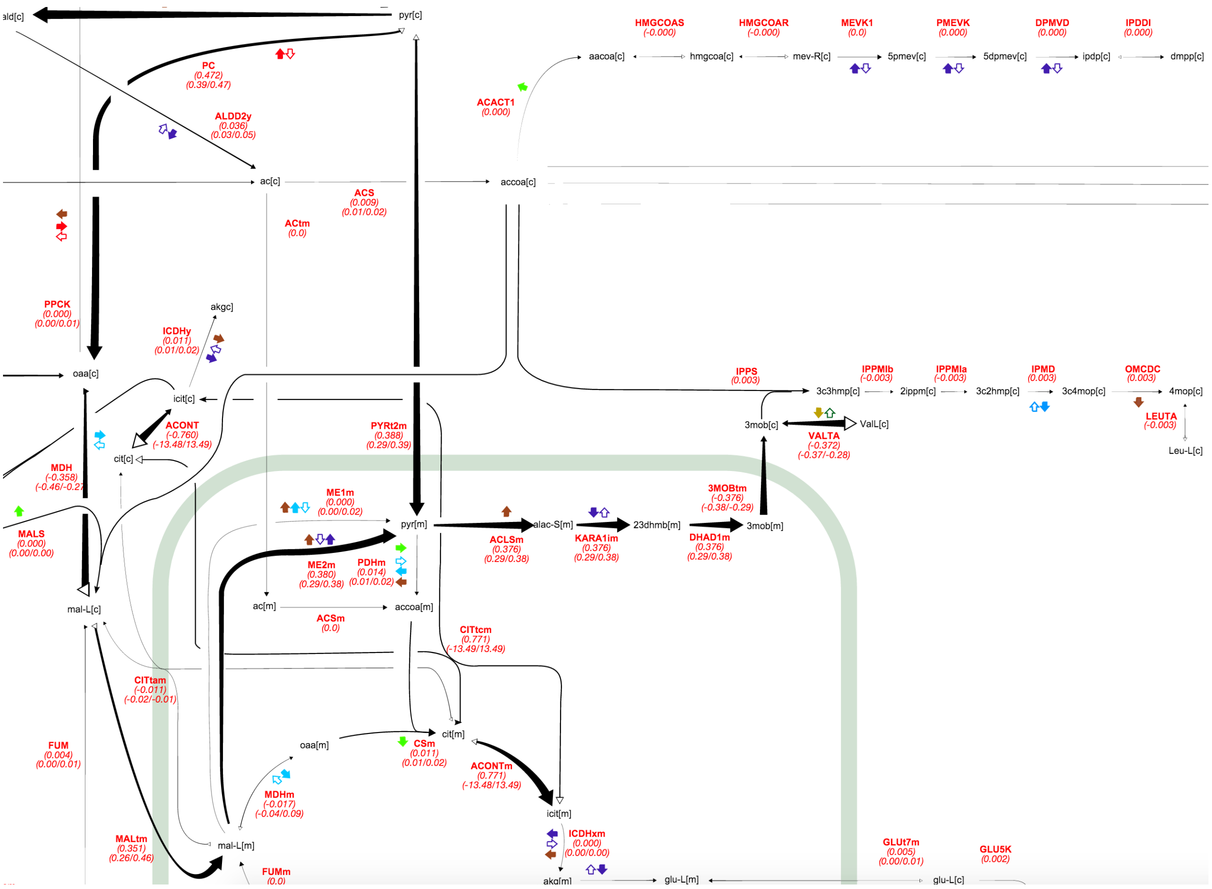

Figure S11. Mitochondrial import and branched-chain amino acid fluxes for base in 2% glucose + 0.2% galactose. For a description of colorful cofactor arrows see the Fits and ELVA plots subsection in the Results section.

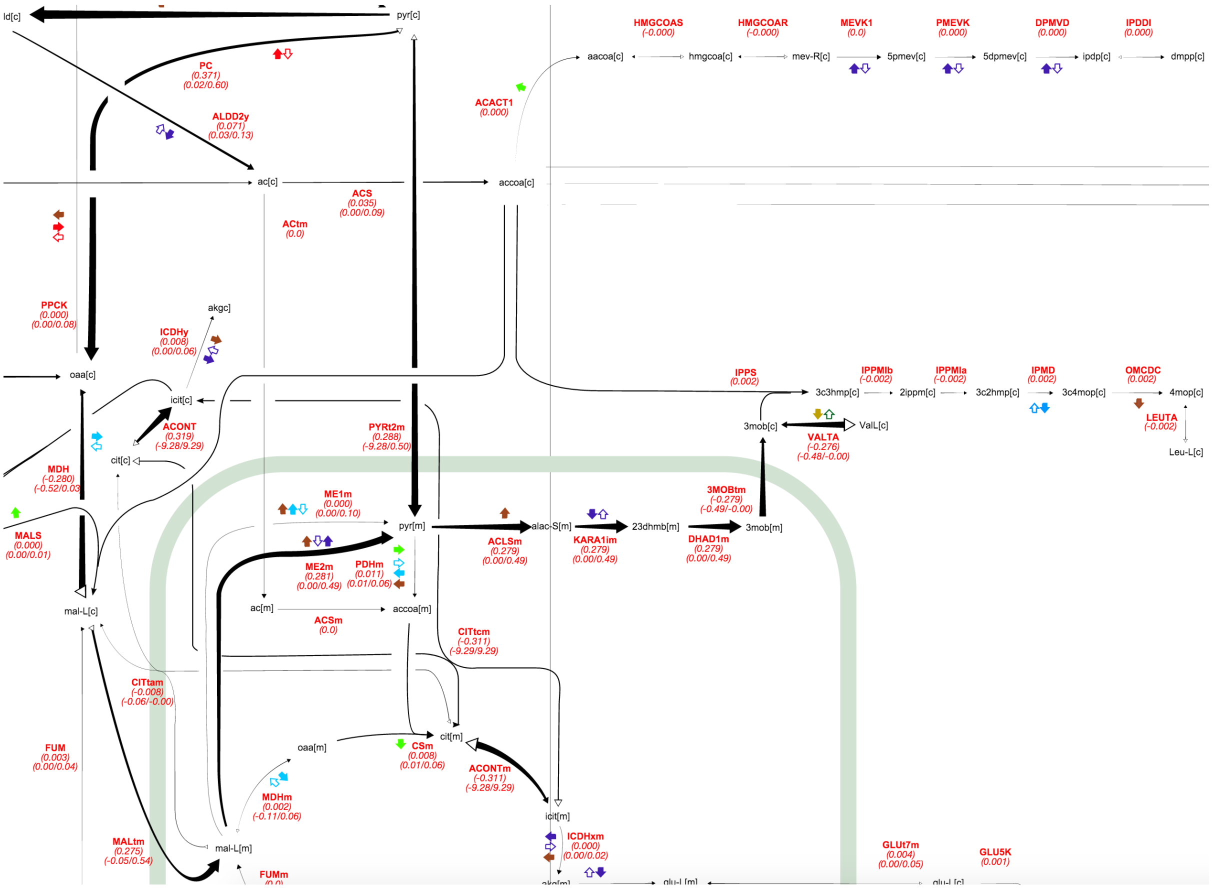

Figure S12. Mitochondrial import and branched-chain amino acid fluxes for sip1Δ in 2% glucose + 0.2% galactose. For a description of colorful cofactor arrows see the Fits and ELVA plots subsection in the Results section.
