# Supplementary material for: Flux-Enabled Exploration of the Role of Sip1 in Galactose Yeast Metabolism
Source: Front Bioeng Biotechnol. 2017 May 24;5:31. doi: 10.3389/fbioe.2017.00031 (PMC5443151; doi:10.3389/fbioe.2017.00031)
Supplement: Additional file 3 — Example extracellular flux calculation and derivation of formula. This is the.html file corresponding to the Jupyter notebook found in Additional file 2 that demonstrates the derivation of the formula used to calculate extracellular fluxes and an example of its use. [file Data_Sheet_3.ZIP › additional_file_3.html]

extracellular\_flux\_calculation\_example


# Exchange flux calculation demonstration¶

Here we demonstrate how to calculate an extracellular flux from raw data.

## Equation¶

Here's the equation used to calculate the extracellular fluxes:

$$
\begin{equation}
\nu\_p = 1000\frac{\mu}{M\_p\alpha}\frac{d\overline{C}p}{dOD}
\end{equation}
$$

$M\_p$ is the corresponding molecular weight of metabolite $p$, $\alpha$ is the conversion factor between OD$\_{600}$ and cell mass concentration in grams of dry cell weight per liter (gDcW/L), and $d\overline{C}p/dOD$ is the slope of the concentration of metabolite $p$ versus OD$\_{600}$.
The value of $\alpha$ was taken to be 0.7742 based on multiple in-house experiments (data not shown).

The desired units are mmol/gDcW/h, where gDcW stands for "grams of dry cell weight". Here is a comparison of the unit conversions as a sanity check:

## Derivation¶

The exchange fluxes $\nu\_p$ for metabolite $p$ are given by the rate of change of metabolites in the system:

$$
\begin{equation}
\nu\_p = \frac{dX\_p}{dt}
\end{equation}
$$

where $X\_p$ represents the total amount (in grams) of metabolite $p$. However, since the more cells you have in the system the higher these exchange fluxes are, they are normalized by the total number of cells in terms of the grams of cell dry weight (gDcW):

$$
\begin{equation}
\nu\_p = \frac{1}{gDcW}\frac{dX\_p}{dt}
\end{equation}
$$

and are expressed in number of molecules (mmol actually)

$$
\begin{equation}
\nu\_p = \frac{1000}{M\_p}\frac{1}{gDcW}\frac{dX\_p}{dt}
\end{equation}
$$

where $M\_p$ is the corresponding molecular weight for metabolite $p$.

Now, this equation can be expressed in terms of metabolite concentration($\overline{C}\_p = X\_p/V$) by dividing by the volume($V$):

$$
\begin{equation}
\nu\_p = \frac{1000}{M\_p}\frac{1}{gDcW/V}\frac{d\overline{C}\_p}{dt}
\end{equation}
$$

and the derivative $d\overline{C}\_p/dt$ can be expressed as a chain of derivatives:

$$
\begin{equation}
\frac{d\overline{C}\_p}{dt} = \frac{d\overline{C}\_p}{dOD\_{600}}\frac{dOD\_{600}}{dgDcW}\frac{dgDcW}{dt}
\end{equation}
$$

yielding:

$$
\begin{equation}
\nu\_p = \frac{1000}{M\_p}\frac{1}{gDcW/V}\frac{d\overline{C}\_p}{dOD\_{600}}\frac{dOD\_{600}}{dgDcW/V}\frac{dgDcW/V}{dt}
\end{equation}
$$

Now, $OD\_{600}$ if proportional to cell density: $gDcW/V = \alpha OD\_{600}$, and gDcW is proportional to the total number of cells N: $gDcW = k N$ so

$$
\begin{equation}
\nu\_p = \frac{1000}{M\_p}\frac{d\overline{C}\_p}{dOD\_{600}}\frac{1}{\alpha}\frac{1}{N}\frac{N}{dt}
\end{equation}
$$

and, assuming the usual expression for growth rate $\mu = 1/N \, dN/dt$, we get:

$$
\begin{equation}
\nu\_p = \frac{1000}{M\_p}\frac{\mu}{\alpha}\frac{d\overline{C}\_p}{dOD\_{600}}
\end{equation}
$$

The units for exchange fluxes are mmol/gDcW/h, where gDcW stands for "grams of dry cell weight". Here is a unit check:

$$
\begin{matrix}
\nu\_p & = & 1000 &\frac{\left [\mu \right]}{\left[ M\_p \right]\left[\alpha\right]}& \left[\frac{d\overline{C}p}{dOD}\right]\\
\left[\frac{mmol}{gDcW \cdot h}\right] & = & \left[\frac{mmol}{mol}\right] & \frac{\left[1/h\right]}{\left[g/mol\right] \left[gDcW/L/OD\_{600}\right]} & \frac{\left[g/L\right]}{\left[OD\_{600}\right]}
\end{matrix} \\\\
$$

## Example: Glucose flux for strain/condition 'U' in flask 1¶

Here we calculate the glucose flux for strain/condition U for flask 1.

First, we need the specific growth rate of the flask.

### Specific growth rate calculation¶

#### Prepare cell concentration time curve data and preview¶

In [1]:

```
# Import dependencies
import sqlite3 as lite
import pandas as pd

# Define columns of Pandas DataFrame
columns = ['StrainId','DateTime','FlaskNum','Species','Concentration']

# Create list of data
df_cell = [[u'U', u'2014-09-02 09:32', 1, u'Cell', 0.2086],
           [u'U', u'2014-09-02 11:40', 1, u'Cell', 0.4842],
           [u'U', u'2014-09-02 12:05', 1, u'Cell', 0.6004],
           [u'U', u'2014-09-02 12:18', 1, u'Cell', 0.663],
           [u'U', u'2014-09-02 12:32', 1, u'Cell', 0.719],
           [u'U', u'2014-09-02 12:46', 1, u'Cell', 0.7678],
           [u'U', u'2014-09-02 13:00', 1, u'Cell', 0.833],
           [u'U', u'2014-09-02 13:14', 1, u'Cell', 0.8986]]

# Convert list to Pandas dataframe of desired data
cell_concns = pd.DataFrame(df_cell,columns=columns)
```

In [2]:

```
cell_concns
```

Out[2]:

|  | StrainId | DateTime | FlaskNum | Species | Concentration |
| --- | --- | --- | --- | --- | --- |
| 0 | U | 2014-09-02 09:32 | 1 | Cell | 0.2086 |
| 1 | U | 2014-09-02 11:40 | 1 | Cell | 0.4842 |
| 2 | U | 2014-09-02 12:05 | 1 | Cell | 0.6004 |
| 3 | U | 2014-09-02 12:18 | 1 | Cell | 0.6630 |
| 4 | U | 2014-09-02 12:32 | 1 | Cell | 0.7190 |
| 5 | U | 2014-09-02 12:46 | 1 | Cell | 0.7678 |
| 6 | U | 2014-09-02 13:00 | 1 | Cell | 0.8330 |
| 7 | U | 2014-09-02 13:14 | 1 | Cell | 0.8986 |

In [3]:

```
%matplotlib inline
import numpy as np

# Get minimum DateTime
min_time = pd.to_datetime(cell_concns['DateTime']).min()

# Calculate elapsed times
cell_concns['elapsed_time'] = cell_concns['DateTime'].apply(lambda x: (pd.to_datetime(x)-min_time).total_seconds()/60.0/60.0)

# Calculate natural log of OD (lnOD) and save 
cell_concns['lnOD'] = np.log(cell_concns['Concentration'])

# Visualize
cell_concns.plot(x='elapsed_time',y='lnOD',style='ro')
```

Out[3]:

```
<matplotlib.axes._subplots.AxesSubplot at 0x10c9015d0>
```

Let's also look at the OD vs time curve:

In [4]:

```
cell_concns.plot(x='elapsed_time',y='Concentration',style='ro')
```

Out[4]:

```
<matplotlib.axes._subplots.AxesSubplot at 0x10bda3750>
```

I tend to find that cells are in exponential phase from 0.25 to 0.9 OD. So I'm going to use point indices 1 to 7 to calculate the slope of lnOD vs time to obtain the specific growth rate.

#### Fit line to data to obtain maximum specific growth rate and visualize¶

In [5]:

```
from sklearn.linear_model import LinearRegression

# Set chosen indices
chosen_point_inds = [1,7]

# Initialize estimator
estimator = LinearRegression()

# Obtain input data
X = cell_concns['elapsed_time'][chosen_point_inds[0]:chosen_point_inds[1]+1].values

# Reshape for regressor input specifications
X = X.reshape([X.shape[0],1])

# Obtain output
y = cell_concns['lnOD'][chosen_point_inds[0]:chosen_point_inds[1]+1]

# Fit estimator
estimator.fit(X,y)

# Get specific growth rate
mu =  estimator.coef_[0]
y_int = estimator.intercept_

# Print line parameters
print 'Slope: %1.4f'%(mu)
print 'Y-intercept: %1.4f'%(y_int)
```

```
Slope: 0.3837
Y-intercept: -1.5044
```

Hence, the maximum specific growth rate for strain/condition 'U' and flask 1 is ~0.3837.

Simulate fitted line:

In [6]:

```
min_time = cell_concns['elapsed_time'].min()
max_time = cell_concns['elapsed_time'].max()

buffer_time = 0.1*(max_time-min_time)

time_points = np.arange(min_time,max_time+buffer_time,0.1)
fitted_concentrations = mu*time_points+y_int
```

Visualize:

In [7]:

```
import matplotlib.pyplot as plt

fig, ax = plt.subplots()

ax.plot(cell_concns['elapsed_time'],cell_concns['lnOD'],'ro',label='data',ms=6)

ax.plot(cell_concns['elapsed_time'][1:],cell_concns['lnOD'][1:],'o',ms=14, 
        markerfacecolor="None",markeredgecolor='red', markeredgewidth=1,label='chosen')

ax.plot(time_points,fitted_concentrations,'r--',label='fit: %1.3f*t+%1.3f'%(mu,y_int))

plt.xlabel("Elapsed time [h]")
plt.ylabel("lnOD")

ax.legend(loc='best')

plt.title('U1: Specific growth rate')

plt.show()
```

### Glucose versus OD slope calculation¶

We also need the slope of the glucose concentration versus OD for the flux calculation.

#### Prepare gluocse data¶

In [9]:

```
# Make list of glucose data corresponding to same flask
df_glucose = [[u'U', u'2014-09-02 11:40', 1, u'Glucose', 22.23994],
[u'U', u'2014-09-02 12:05', 1, u'Glucose', 21.23914],
[u'U', u'2014-09-02 12:18', 1, u'Glucose', 25.91575],
[u'U', u'2014-09-02 12:32', 1, u'Glucose', 24.53842],
[u'U', u'2014-09-02 12:46', 1, u'Glucose', 23.01908],
[u'U', u'2014-09-02 13:00', 1, u'Glucose', 20.37326],
[u'U', u'2014-09-02 13:14', 1, u'Glucose', 19.44218]]

# Convert to Pandas DataFrame
glucose_concns = pd.DataFrame(df_glucose,columns=columns)
```

In [10]:

```
glucose_concns
```

Out[10]:

|  | StrainId | DateTime | FlaskNum | Species | Concentration |
| --- | --- | --- | --- | --- | --- |
| 0 | U | 2014-09-02 11:40 | 1 | Glucose | 22.23994 |
| 1 | U | 2014-09-02 12:05 | 1 | Glucose | 21.23914 |
| 2 | U | 2014-09-02 12:18 | 1 | Glucose | 25.91575 |
| 3 | U | 2014-09-02 12:32 | 1 | Glucose | 24.53842 |
| 4 | U | 2014-09-02 12:46 | 1 | Glucose | 23.01908 |
| 5 | U | 2014-09-02 13:00 | 1 | Glucose | 20.37326 |
| 6 | U | 2014-09-02 13:14 | 1 | Glucose | 19.44218 |

#### Obtain glucose concentration versus OD data¶

In [11]:

```
cell_concns
```

Out[11]:

|  | StrainId | DateTime | FlaskNum | Species | Concentration | elapsed\_time | lnOD |
| --- | --- | --- | --- | --- | --- | --- | --- |
| 0 | U | 2014-09-02 09:32 | 1 | Cell | 0.2086 | 0.000000 | -1.567337 |
| 1 | U | 2014-09-02 11:40 | 1 | Cell | 0.4842 | 2.133333 | -0.725257 |
| 2 | U | 2014-09-02 12:05 | 1 | Cell | 0.6004 | 2.550000 | -0.510159 |
| 3 | U | 2014-09-02 12:18 | 1 | Cell | 0.6630 | 2.766667 | -0.410980 |
| 4 | U | 2014-09-02 12:32 | 1 | Cell | 0.7190 | 3.000000 | -0.329894 |
| 5 | U | 2014-09-02 12:46 | 1 | Cell | 0.7678 | 3.233333 | -0.264226 |
| 6 | U | 2014-09-02 13:00 | 1 | Cell | 0.8330 | 3.466667 | -0.182722 |
| 7 | U | 2014-09-02 13:14 | 1 | Cell | 0.8986 | 3.700000 | -0.106917 |

First, we merge the data at the same time points:

In [12]:

```
# Merge data at DateTimes
od_concn = pd.merge(cell_concns[['DateTime','Concentration']],
         glucose_concns[['DateTime','Concentration']],
         how='inner',on='DateTime')

# Rename concentrations to reflect either OD or the glucose concentration
od_concn.rename(columns={'Concentration_x': 'OD',
                  'Concentration_y': 'Concentration'}, inplace=True)

# Preview result
od_concn
```

Out[12]:

|  | DateTime | OD | Concentration |
| --- | --- | --- | --- |
| 0 | 2014-09-02 11:40 | 0.4842 | 22.23994 |
| 1 | 2014-09-02 12:05 | 0.6004 | 21.23914 |
| 2 | 2014-09-02 12:18 | 0.6630 | 25.91575 |
| 3 | 2014-09-02 12:32 | 0.7190 | 24.53842 |
| 4 | 2014-09-02 12:46 | 0.7678 | 23.01908 |
| 5 | 2014-09-02 13:00 | 0.8330 | 20.37326 |
| 6 | 2014-09-02 13:14 | 0.8986 | 19.44218 |

#### Visualize¶

In [13]:

```
od_concn.plot(x='OD',y='Concentration',style='ro')
```

Out[13]:

```
<matplotlib.axes._subplots.AxesSubplot at 0x10458e4d0>
```

There appears to be a linear portion corresponding to point indices 2 to 6.

#### Fit line and obtain slope¶

In [14]:

```
from sklearn.linear_model import LinearRegression

# Initialize estimator
estimator = LinearRegression()

# Set chosen indices
chosen_point_inds = [2,6]

# Obtain input data
X = od_concn['OD'][chosen_point_inds[0]:chosen_point_inds[1]+1].values

# Reshape for regressor input specifications
X = X.reshape([X.shape[0],1])

# Obtain output
y = od_concn['Concentration'][chosen_point_inds[0]:chosen_point_inds[1]+1]

# Fit estimator
estimator.fit(X,y)

# Get specific growth rate
dCpdOD =  estimator.coef_[0]
od_concn_y_int = estimator.intercept_

# Print line parameters
print 'Slope: %1.4f'%(dCpdOD)
print 'Y-intercept: %1.4f'%(od_concn_y_int)
```

```
Slope: -29.1800
Y-intercept: 45.3096
```

So, the slope of the glucose concentration versus OD curve is -29.1800 g/L/OD.

#### Visualize data, picked points, and fit line¶

In [15]:

```
# Obtain minimum and maximum OD
min_OD = od_concn['OD'].min()
max_OD = od_concn['OD'].max()

# Calculate buffer zone
buffer_time = 0.1*(max_OD-min_OD)

# Obtain fit line data
OD_points = np.arange(min_OD,max_OD+buffer_time,0.1)
fitted_concentrations = dCpdOD*OD_points+od_concn_y_int
```

In [16]:

```
import matplotlib.pyplot as plt

fig, ax = plt.subplots()

ax.plot(od_concn['OD'],od_concn['Concentration'],'ro',label='data',ms=6)

ax.plot(od_concn['OD'][chosen_point_inds[0]:chosen_point_inds[1]+1],
        od_concn['Concentration'][chosen_point_inds[0]:chosen_point_inds[1]+1],
        'o',ms=14,markerfacecolor="None",markeredgecolor='red', 
        markeredgewidth=1,label='chosen')

ax.plot(OD_points,
        fitted_concentrations,
        'r--',
        label='fit: %1.3f*t+%1.3f'%(dCpdOD,od_concn_y_int))

plt.xlabel("OD")
plt.ylabel("metabolite concentration")

ax.legend(loc='best')

plt.title('U1: dCpdOD')

plt.show()
```

#### Flux calculation¶

Now that we have the specific growth rate and the glucose concentraiton versus OD slope we can calculate the corresponding glucose flux.

The moleculuar weight of glucose is 180.1559 g/mol, the

Finally, we calculate the flux using the values:

$$
\begin{equation}
\nu\_{glucose} = 1000\frac{\mu}{M\_p\alpha}\frac{d\overline{C}p}{dOD}\\
\nu\_{glucose} = 1000 \left[\frac{mmol}{mol}\right] \frac{0.38371046557 \left[1/h\right]}{180.1559 \left[g/mol\right] 0.7742 \left[gDcW/L/OD\_{600}\right]}\left[-29.1800183258\right]\frac{\left[g/L\right]}{\left[OD\_{600}\right]}\\
= -80.2763371613131 \left[\frac{mmol}{gDcW\*h}\right]
\end{equation}
$$

Here's the Python calculation:

In [17]:

```
-1000*0.38371046557/180.1559/0.7742*(29.1800183258)
```

Out[17]:

```
-80.2763371613131
```

Thus, the glucose consumption flux for flask 1 of strain/condition U is about -80.28 mmol/gDcW/h.
